# Protease-assisted microencapsulation of carvacrol in pea protein systems for enhanced and durable antibiofilm activity

**DOI:** 10.1016/j.crfs.2026.101522

**Published:** 2026-08-05

**Authors:** Jun Ji, Nour-Eddine Chihib, Géraldine Agusti, Emilie Dumas, Adem Gharsallaoui

**Affiliations:** a Université Lyon 1, CNRS, LAGEPP, UMR 5007, Bourg-en-Bresse, France; b University of Lille, CNRS, INRAE, Centrale Lille, UMR 8207-Unité Matériaux et Transformations, Lille, 59120, France

**Keywords:** Carvacrol, Protein-based microcapsules, Interfacial engineering, Protease-assisted delivery, Antibiofilm activity, Bio-derived materials

## Abstract

Developing dry antimicrobial delivery systems that combine bioactive protection with biofilm-matrix disruption remains a major challenge. Building on a previously established protease-modulated pea protein isolate (PPI)-carvacrol nanoemulsion system, this study developed protease-assisted microcapsules designed to preserve carvacrol and promote the removal of preformed biofilms. Carvacrol-loaded nanoemulsions were prepared at pH 3.5, 7.0, and 10.0 and supplemented with pepsin or trypsin before spray-drying or freeze-drying with maltodextrin.

Protease-specific interfacial modification markedly affected emulsion stability, powder structure, and encapsulation performance. At pH 7.0 and 10.0, trypsin reduced droplet size from 284.60 to 232.52 nm and from 149.13 to 140.38 nm, respectively. The resulting spray-dried microcapsules exhibited high encapsulation efficiency (>96%) and low surface carvacrol contents (0.95–1.24 mg/g). In contrast, pepsin caused pronounced destabilization under acidic conditions, particularly after freeze-drying, yielding porous powders with an encapsulation efficiency of 48.18% and a surface carvacrol content of 69.51 mg/g.

Trypsin-assisted microcapsules achieved 90–99% removal of preformed *Listeria innocua* biofilm biomass within 1 h, whereas enzyme-free and pepsin-containing formulations generally remained below 40%. Microscopic observations confirmed extensive disruption and detachment of the biofilm structure. After one year at 4 °C, trypsin-loaded formulations retained high biofilm biomass removal activity (85–99% after 2 h).

These findings demonstrate that protease-assisted microencapsulation can couple interfacial regulation during particle formation with enzyme-mediated biofilm-matrix disruption after rehydration, providing a sustainable carvacrol delivery platform with durable antibiofilm functionality.

## Highlights

• Enzyme-modulated interfacial assembly directs microstructure formation.
• Spray-drying yields dense particles while freeze-drying creates porous matrices.
• Structure-property relationships govern encapsulation and release performance.
• Protease-assisted materials achieve rapid eradication of mature biofilms.
• Functional stability is maintained during long-term refrigerated storage.

## Graphical abstract

**Figure:**
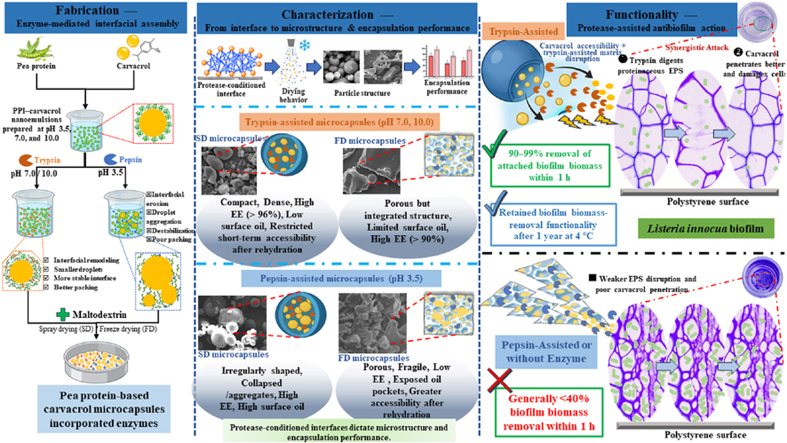

## 1 Introduction

Foodborne pathogens such as *Listeria monocytogenes* and *Escherichia coli* are leading causes of foodborne illness and pose severe public health concerns. Listeriosis, caused by *L. monocytogenes*, is a relatively rare but severe foodborne disease. In 2023, 2993 confirmed cases were reported in the EU/EEA, corresponding to a notification rate of 0.67 cases per 100,000 population (European Centre for Disease Prevention and Control (European Centre for Disease Prevention and Control, 2025). Importantly, these pathogens readily form biofilms on food-contact surfaces, where they are protected by extracellular polymeric substances (EPS) that render them resistant to sanitizers and preservatives (Osek et al., 2022). In food processing environments, bacterial biofilms on conveyor belts, cutting tools, and stainless-steel equipment can lead to persistent contamination and repeated outbreaks. Conventional chemical preservatives (e.g. benzoates, nitrates, sorbates) and disinfectants may reduce planktonic pathogens but often fail to penetrate biofilms and may carry health or regulatory concerns. Indeed, many of these synthetic preservatives have been linked to adverse health effects (carcinogenic, allergenic) and are falling out of favor (Shi et al., 2024). Consumers therefore now prefer “clean label” foods with natural antimicrobials. Thus, there is an urgent need for safer, more sustainable antimicrobial strategies capable of eradicating biofilm-embedded pathogens on foods and processing surfaces.

Plant essential oils (EOs) are promising natural antimicrobials for food safety applications. Many EO constituents, such as phenolic monoterpenes, exert broad-spectrum antibacterial effects through multiple mechanisms (Xu et al., 2024). Carvacrol, a major component of oregano and thyme oils, is widely noted for potent antibacterial, antifungal, and antiseptic activity. It targets microbial membranes (due to its phenolic hydroxyl) and can generate reactive oxygen species, causing membrane perturbation, ATP leakage and cell death (Xu et al., 2024). These actions are largely non-specific, so carvacrol is effective against both Gram-positive and Gram-negative bacteria, including *L. monocytogenes*, *E. coli* and *Salmonella*. In fact, studies show carvacrol can inhibit biofilm formation and even disrupt mature biofilms of *L. monocytogenes* on stainless steel and other surfaces (Yammine et al., 2023a, 2024). As EOs are generally regarded as safe and possess antioxidant properties, they fit well within the ‘clean-label’ trend in food preservation. A key limitation of EOs is their strong volatility and poor water solubility. Carvacrol and similar compounds evaporate quickly and can oxidize or lose efficacy during storage, processing or on surfaces (Sousa et al., 2022). Their strong odor and low dispersibility in aqueous foods further restrict direct use. These issues have motivated interest in delivery technologies: encapsulation and nanoemulsions can retain volatile EOs, mask odors, improve solubility, and control release. For example, spray-dried microcapsules of carvacrol have shown enhanced stability and biofilm-removal potency in model systems (Mechmechani et al., 2022b). However, current carvacrol delivery systems may still suffer from core-material migration, surface-oil accumulation, premature or burst release, and loss of active compounds during drying and prolonged storage. For example, encapsulation carrier design has been shown to strongly affect the physicochemical, thermal, antimicrobial, and release properties of EO nanoparticles (Kaboudi et al., 2023). Moreover, most existing systems primarily focus on protecting and releasing carvacrol and do not actively disrupt the EPS matrix that limits its penetration into mature biofilms or maximize its bactericidal efficacy against embedded cells. Also, co-delivery strategies combining carvacrol with EPS-disrupting agents and their long-term functional stability remain insufficiently explored. Therefore, developing an effective delivery system that integrates carvacrol retention, biofilm-matrix disruption, and durable functionality remains an important challenge.

Proteins from renewable plant sources like pea (*Pisum sativum*) offer attractive wall materials for encapsulating labile volatiles. Pea protein isolates form strong gels and bind hydrophobic actives stably, making them sustainable and effective carriers for bioactives (Oliveira et al., 2025). Its major globulin fractions, legumin and vicilin, can adsorb at oil–water interfaces and stabilize hydrophobic carvacrol droplets, while their interfacial organization can be altered by controlled proteolysis. Previous work has also demonstrated that structural modification of proteins, combined with maltodextrin, can influence the formation and performance of spray-dried microcapsules (Younesi et al., 2023). Microencapsulation of carvacrol in protein matrices and combined with maltodextrin has been shown to boost its bioactivity. For example, spray-dried carvacrol maltodextrin capsules (caseinate-based) had a 4-fold lower MIC against *Pseudomonas aeruginosa* than free carvacrol and achieved significant biofilm reductions (Mechmechani et al., 2022b). Likewise, nanoencapsulated carvacrol completely eradicated *Salmonella* Enteritidis biofilms in 15 min at moderate doses (Yammine et al., 2023b). Nevertheless, even encapsulated EOs can fail to fully disrupt mature biofilms under all environmental conditions, so complementary biofilm-eradication strategies are needed. In most such studies, enzymatic hydrolysis is applied prior to emulsification to modify protein structure and thereby improve the functional properties of the carrier matrix and encapsulation efficiency (EE) of EOs/actives. In contrast, the present system incorporated active proteases directly into pre-formed PPI-stabilized carvacrol nanoemulsions, where they were intended not only to remodel the protein-stabilized interfacial layer but also to contribute biological functionality through enzyme-assisted disruption of biofilm EPS components. This strategy therefore combines interfacial engineering and antibiofilm activity within a single formulation, while allowing both functions to be retained during subsequent drying and storage.

Proteolytic enzymes are effective biofilm-dispersing agents because they hydrolyze the protein-rich EPS matrix (Wang et al., 2023). Trypsin, a pancreatic serine protease active under neutral to alkaline pH conditions, can degrade mature biofilms formed by both Gram-negative and Gram-positive bacteria, although when used alone it typically results in only partial biomass reduction. Similarly, papain, a cysteine protease, has been reported to cleave protein components of the biofilm matrix (Wang et al., 2023). These proteases can hydrolyze these extracellular proteins, disrupt the EPS scaffold, and render biofilms and bacterial cells more susceptible to antimicrobials. Crucially, proteases can act synergistically with antimicrobials. For example, co-treatment with trypsin or pepsin and carvacrol completely dispersed mature *P. aeruginosa* and *Enterococcus faecalis* biofilms (Mechmechani et al., 2022a). Indeed, co-applying protease can weaken biofilm structure and enable deeper penetration of antimicrobial agents like carvacrol, thereby suggesting a clear rationale for co-encapsulation of a protease and carvacrol. In this study, pepsin and trypsin were incorporated separately as proteases with contrasting pH-activity profiles, with pepsin representing acidic proteolysis and trypsin representing neutral–alkaline proteolysis, and proteinaceous EPS degradation. Recent studies have confirmed their antibiofilm potential. For example, the sequential treatment by microcapsules containing trypsin, pepsin, and carvacrol was shown to eradicate biofilms more efficiently, and reduce *P. aeruginosa* by 5 log CFU mL^−1^ and *Enterococcus faecalis* by 4 log CFU mL^−1^ (Mechmechani et al., 2022a). Our previous study also systematically established that pepsin and trypsin exerted distinct pH-dependent effects on PPI–carvacrol nanoemulsion stability and antibiofilm functionality (Ji et al., 2025). Specifically, trypsin promoted moderate interfacial remodeling and enhanced antibiofilm activity at neutral and alkaline pH, whereas pepsin caused marked interfacial destabilization under acidic conditions. The present work therefore does not seek to re-establish these interfacial mechanisms, but uses them as the mechanistic basis for microcapsule design.

Nevertheless, it remains unclear whether nanoemulsions containing active proteases can be converted into dry powders while preserving favorable encapsulation properties and protease-assisted antibiofilm functionality. In particular, the effects of spray-drying and freeze-drying on protease-conditioned interfaces, carvacrol migration, powder architecture, and long-term biological performance have not been systematically connected. Addressing this gap is necessary to determine whether the previously developed liquid nanoemulsion system can be translated into a practical, storage-stable antimicrobial platform. Such a “dual-action” formulation would capitalize on pea protein's protective matrix, while enzymatically breaking down the biofilm matrix, simultaneously releasing the volatile carvacrol, maintaining prolonged contact, thus greatly enhancing antibiofilm efficacy and delivery stability.

In this study, building on our previous investigation of protease-mediated interfacial behavior in PPI-stabilized carvacrol nanoemulsions, we developed a protease-assisted pea-protein microencapsulation strategy to convert these bioactive nanoemulsions into storage-stable dry powders suitable for practical antibiofilm applications. Pepsin or trypsin was integrated into pre-formed carvacrol-loaded nanoemulsions prior to drying to create multifunctional microcapsules capable of both antimicrobial delivery and biofilm-matrix disruption. We hypothesized that enzyme- and pH-dependent interfacial states would influence microcapsule formation and encapsulation performance, while the retained protease would subsequently also contribute to biofilm disruption after rehydration. The pH-dependent effects of the two proteases on the feed emulsions were characterized to establish their relationship with key physicochemical properties of microcapsules produced by either spray-drying or freeze-drying, including moisture content, hygroscopicity, particle size, morphology, and encapsulation behavior, together with FTIR analyses to elucidate structural interactions. Antibacterial activity against *L. innocua* and *E. coli*, and antibiofilm efficacy against preformed *L. innocua* biofilms on polystyrene surfaces were further evaluated, together with the retention of biological activity during extended storage, thereby establishing a mechanistic link between protease-mediated interfacial engineering and functional antimicrobial performance. Overall, this study constructed a protease-assisted EO microencapsulation framework that provided a theoretical and technological basis for designing durable, long-acting antimicrobial delivery systems suitable for food preservation and treatment of food-contact surfaces, offering a scalable, bio-derived solution to persistent contamination and biofilm-related challenges in modern food safety.

## 2 Materials and methods

### 2.1 Materials

Trypsin from porcine pancreas (1000–2000 BAEE units/mg solid; EC number 232-650-8) and imidazole (≥99.5%) were purchased from Sigma-Aldrich (St. Louis, MO, USA). Pepsin A from porcine gastric mucosa (activity ≥1:10,000, according to the supplier's specification; EC number 232-629-3) was obtained from MP Biomedicals (Strasbourg, France). Carvacrol (≥98% purity) was purchased from Sigma Aldrich (St. Louis, MO, USA), with a molecular weight of 150.22 g/mol and a density of 0.976 g/cm^3^. Pea protein isolate (≥84% protein) and maltodextrins DE 19 were purchased from Roquette-Frères SA (Lestrem, France). All other chemicals were of analytical reagent grade and used without further purification. Distilled water was used throughout the experiment.

### 2.2 Bacterial strains and culture conditions

*Listeria innocua* (ATCC 33090) strain and *Escherichia coli* (ATCC 10536) strain were used in this study. The bacteria strains were stored at −80 °C in Tryptone Soy Broth (TSB; Biokar Diagnostics, Pantin, France) supplemented with 20% (v/v) glycerol. Microbial culture was performed based on the previous methods (Liao et al., 2021). Prior to experiments, one mL of each stock strain was transferred into 9 mL of TSB for 8 h at 37 °C, then one mL of this pre-culture was transferred into a new 9 mL aliquot of TSB and incubated at 37 °C for 16 h. Following exponential growth, the pre-cultured bacteria were diluted in TSB to a final concentration of 10^6^ CFU/mL for the antimicrobial and biofilm formation assays.

### 2.3 Microcapsules formation *via* spray-drying and freeze-drying techniques

#### 2.3.1 Preparation of nanoemulsions

Pea protein isolate (4 g) was dispersed in distilled water and hydrated overnight at room temperature. During hydration, the pH was adjusted to 3.5, 7.0, or 10.0 using HCl or NaOH solutions (0.1 or 1.0 M). The hydrated protein dispersions were then centrifuged at 12,000 rpm for 10 min to remove the insoluble protein fraction. Carvacrol (20 g) was added to the recovered protein dispersion, and the mixture was homogenized using an Ultra-Turrax PT 4000 homogenizer (Polytron, Kinematica, Switzerland) at 14,000 rpm for 5 min at room temperature to obtain a coarse oil-in-water emulsion. The coarse emulsion was subsequently processed using an LM20 Microfluidizer (Microfluidics Co., MA, USA) by applying two passes at 500 bar followed by one pass at 1000 bar. After microfluidization, the pH of each fine emulsion was readjusted to its target value of 3.5, 7.0, or 10.0.

Freshly prepared aqueous pepsin or trypsin solutions, each containing 0.4 g of enzyme on a dry-mass basis and adjusted to the corresponding formulation pH, were slowly added to the fine emulsions. The pH was maintained at the target value by titration with HCl or NaOH, and the formulations were mixed for 15 min. Protease-free emulsions prepared under the same conditions were used as formulation controls. This procedure yielded nine feed emulsions corresponding to three enzyme conditions (no enzyme, pepsin, or trypsin) at each of the three pH values.

A maltodextrin DE 19 stock solution was then gradually added to each emulsion while maintaining the target pH, and the formulations were mixed for an additional 30 min before drying. All concentrations were expressed on a total wet-formulation mass basis before drying. Each formulation had a final mass of 400 g and contained 1% (w/w) pea protein isolate, 5% (w/w) carvacrol, 20% (w/w) maltodextrin DE 19, and either 0.1% (w/w) pepsin, 0.1% (w/w) trypsin, or no enzyme, with distilled water accounting for the remaining mass.

#### 2.3.2 Spray-drying and freeze-drying processes

The above freshly prepared emulsion compositions were divided into two equal parts, one for spray-drying and one for freeze-drying. The feed emulsions for spray-drying were injected into a laboratory spray-dryer equipped with a 0.5 mm nozzle atomizer (Mini Spray-Dryer Büchi B-290, Switzerland) under stirring at 200 rpm. The operational conditions used for the spray-drying process were as follows: air pressure 4-5 bar, inlet air temperature 180 ± 2 °C, outlet air temperature 100 ± 10 °C, and feed flow rate 0.5 L/h. The other emulsion compositions were directly placed at −20 °C for 48 h and subsequently freeze-dried (BUCHI Lyovapor™ L-200, Switzerland) for 72–96 h at a condenser temperature of −58 ± 2 °C and a chamber pressure of 0.08 mbar. The formed microcapsule powders were collected in sealed containers, and weighed to calculate the encapsulation yield, and then stored at 4 °C until analysis. Table 1 shows the abbreviations of different microcapsule samples.

**Table 1:** Abbreviations for protease-assisted pea protein-based carvacrol microcapsule powders.

Abbreviations	pH of pea protein O/W emulsion	Incorporated enzymes	Drying method
SN7	7.0	No enzyme	Spray-drying
FN7	7.0	No enzyme	Freeze-drying
SP7	7.0	Pepsin	Spray-drying
FP7	7.0	Pepsin	Freeze-drying
ST7	7.0	Trypsin	Spray-drying
FT7	7.0	Trypsin	Freeze-drying
SN3.5	3.5	No enzyme	Spray-drying
FN3.5	3.5	No enzyme	Freeze-drying
SP3.5	3.5	Pepsin	Spray-drying
FP3.5	3.5	Pepsin	Freeze-drying
ST3.5	3.5	Trypsin	Spray-drying
FT3.5	3.5	Trypsin	Freeze-drying
SN10	10.0	No enzyme	Spray-drying
FN10	10.0	No enzyme	Freeze-drying
SP10	10.0	Pepsin	Spray-drying
FP10	10.0	Pepsin	Freeze-drying
ST10	10.0	Trypsin	Spray-drying
FT10	10.0	Trypsin	Freeze-drying

Note: S and F represent spray-drying and freeze-drying, respectively; N, P, and T represent no enzyme, pepsin, and trypsin, respectively; the numbers 3.5, 7, and 10 indicate the pH of the feed nanoemulsions before drying.

### 2.4 Physicochemical characterization of O/W nanoemulsions and microcapsules

#### 2.4.1 Particle size distribution and surface charge (ζ-potential) of O/W nanoemulsions

The effects of enzyme incorporation on the particle size and surface charge of pea protein-based O/W emulsion at different pH values were evaluated. The size and polydispersity index (PDI) of encapsulated carvacrol oil droplets in the original emulsions as well as the enzyme-incorporated emulsions were determined by dynamic light scattering (DLS) using a Zetasizer Nano-ZS90 (Malvern Instruments Ltd., Worcestershire, UK) at a fixed angle of 90°. Briefly, 0.1 mL of freshly prepared emulsions were added to 10 mL of imidazole-acetate (5 mM) buffer with corresponding pH (3.5, 7.0 or 10.0) and homogenized to eliminate multiple scattering effects. The refractive index of the carvacrol oil droplets used in the light scattering calculations was 1.532 with an absorption of 0.01, and the refractive index of the water phase was 1.33. The analysis was performed after 90 s of equilibrium. All measurements were conducted in triplicate at 25 °C, and the mean sizes were expressed in nanometers (nm).

The ζ-potential of emulsions was measured by electrophoretic mobility using a Ζetasizer Nano-ZS90 (Malvern Instruments Ltd., Worcestershire, UK). To avoid multiple scattering effects, the nanoemulsions were diluted with imidazole-acetate buffer with corresponding pH. All measurements were performed three times and the mean ζ-potential values were calculated by instrument as the average of three individual measurements.

#### 2.4.2 Particle size of microcapsules

The particle size of the dry microcapsule powders was determined *via* low-angle laser scattering using a Malvern Mastersizer 3000 instrument (Malvern Instruments Ltd., Worcestershire, UK). Prior to analysis, the samples were evenly dispersed in absolute ethanol by vortexing. The microcapsule suspensions were then separately injected into the measurement chamber filled with absolute ethanol and continually stirred at 2000 rpm, to avoid multiple scattering effects and obtain homogeneous samples. Each sample was measured at least three times, and the mean particle sizes were expressed as the volume-weighed mean diameter D_4,3_ and the volume-surface mean diameter D_3,2_, providing more comprehensive information on the particle size distribution of spray-dried and freeze-dried microcapsules. The refractive index of maltodextrin was set to 1.67, and that of the ethanol dispersion phase to 1.36. To obtain more information about the particle size distribution, the span values of the microcapsule particle distribution were calculated using the D_10_, D_50_, and D_90_, which represent the diameters corresponding to 10%, 50%, and 90% of the entire particle number or volume, respectively. The span was calculated as follows:

(1) $$Span=\frac{D_{90}-D_{10}}{D_{50}}$$

#### 2.4.3 Surface morphology observation of microcapsules

Scanning electron microscopy (SEM) was performed using an FEI Quanta 250 FEG microscope at the Centre Technologique des Microstructures (CTμ), University of Lyon (Villeurbanne, France). Microcapsule powders were deposited on flat steel stubs and coated under vacuum by cathodic sputtering prior to observation. Images were acquired under high-vacuum conditions at an accelerating voltage of 10.0 kV. Spray-dried samples were observed at a magnification of 3000×, whereas freeze-dried samples were examined at a magnification of 400×.

#### 2.4.4 Moisture content and water activity of microcapsules

The residual moisture content (MC) was determined by weighing 1.00 g of carvacrol microcapsule powder into a disposable aluminum dish, and then drying at 105 °C for 24 h. The moisture content (%) was calculated using the following equation:

(2) $$MC\;\left(\%\right)=\frac{W_{0}-W_{1}}{W_{0}}\times 100$$

where W_0_ is the initial weight of the powder before drying, and W_1_ is the weight of the powder after drying at 105 °C for 24 h.

Furthermore, the weighed microcapsule powder was placed into the measuring chamber of the water activity meter (Novasina AG, Lachen, Swiss), the water activity of the microcapsule was obtained in rapid mode at room temperature. All values shown represent the mean of three independent experiments for each sample.

#### 2.4.5 Hygroscopicity of microcapsules

For the determination of hygroscopicity, each microcapsule powder (approximately 1.00 g) was placed in a Petri dish in a sealed container containing NaCl saturated solution at 23 ± 2 °C and 75-76% relative humidity (RH) (Bajac et al., 2022). Samples were weighed after one week. Hygroscopicity of microcapsules was defined as the grams of water absorbed per 100 g of dry powder.

#### 2.4.6 Encapsulation efficiency, oil retention efficiency, and loading capacity of microcapsules

The encapsulation properties of carvacrol within the freshly prepared microcapsules were evaluated immediately, according to previously described methods (Brahmi et al., 2025b; Sun et al., 2020). The encapsulation characteristics were determined immediately after microcapsule preparation to minimize potential changes in carvacrol distribution caused by storage, migration, or volatilization. Briefly, for the total carvacrol content in microcapsules, 20 mg of microcapsule powder was dispersed in 20 mL of n-hexane with gentle stirring at 700 rpm for 20 min, followed by ultrasonic treatment (Multifunctional Ultrasonic Cleaner, France) at a frequency of 40 kHz and an amplitude of 100% for 1 h. The supernatant was then collected by centrifugation at 10,000 rpm and 4 °C for 10 min. For the determination of surface carvacrol content, 20 mg of microcapsule powder was dispersed in 20 mL of n-hexane and stirred at 700 rpm for 10 min, followed by centrifugation under the same conditions to obtain the supernatant. The collected supernatants were diluted to an appropriate concentration in n-hexane and measured using a UV-visible spectrophotometer at a wavelength of 275 nm, followed by calculation of the carvacrol content using a standard curve. The linearity and repeatability of the carvacrol quantification method were evaluated using a calibration curve prepared in n-hexane over a concentration range of 0.000781–0.1 mg/mL. Absorbance was measured at 275 nm, and each concentration was analyzed in triplicate. The calibration curve was described by the equation (y = 13.155x + 0.0109) and showed excellent linearity, with a coefficient of determination of (R^2^ = 0.9999). Triplicate measurements were used to verify analytical repeatability. However, a complete validation including matrix-spike recovery experiments was not performed in the present study. Encapsulation efficiency is defined as the percentage of retained carvacrol successfully entrapped into the microcapsules, while oil retention efficiency is defined as the percentage of total carvacrol retained (i.e., excluding the oil loss) during the drying process, and oil loading capacity is the amount of carvacrol contained in the obtained microcapsules (Bringas-Lantigua et al., 2011). The encapsulation efficiency (EE), loading capacity (LC) and oil retention efficiency (RE) were calculated using the following equations:

(3) $$EE\;\left(\%\right)=\frac{W_{t}-W_{s}}{W_{t}}\times 100$$

(4) $$LC\;\left(mg/g\right)=\frac{W_{t}-W_{s}}{W_{i}}$$

(5) $$RE\;\left(\%\right)=\frac{W_{t}}{W_{a}}\times 100$$

where W_t_ is the total mass (mg) of carvacrol on/in the microcapsules after encapsulation; W_s_ is the mass (mg) of carvacrol on the surface of microcapsules; W_i_ is the weight of the microcapsules powder used to calculate the total oil and surface oil; W_a_ is the theoretical weight of carvacrol in the microcapsule powder initially added.

#### 2.4.7 Attenuated total reflectance–fourier transform infrared (ATR-FTIR) spectroscopy

The microcapsules were characterized using an FTIR spectrometer equipped with ATR (Nicolet iS50, Thermo Scientific, Waltham, MA, USA). The FTIR spectra of all samples were recorded at a resolution of 4 cm^−1^ with 64 scans in the wavelength range of 4000–400 cm^−1^.

### 2.5 Antimicrobial activities of microcapsules against planktonic bacteria cells

#### 2.5.1 Evaluation of antimicrobial activity *via* agar disk diffusion assay

To evaluate the antimicrobial efficacy of these microcapsules against selected Gram-negative (*E. coli ATCC 10536*) and Gram-positive (*L. innocua ATCC 33090*) foodborne pathogens, agar disk diffusion assays were conducted in accordance with the protocol outlined by Brahmi et al. (2025a). Briefly, prior to the experiment, the microcapsule samples were exposed to an ultraviolet lamp for 40 min to ensure complete sterilization. Then, 100 μL of a bacterial suspension, with a final concentration of 10^6^ CFU/mL, was uniformly spread onto the surface of a TSA agar dish. Subsequently, 30 mg of microcapsule powders were introduced into pre-prepared holes in the agar dishes. The Petri dishes were then incubated at 4 °C for 1 h to facilitate optimal diffusion of the active components within the microcapsules, followed by further incubation at 37 °C for 24 h. The diameters of the resulting inhibition zones were measured in millimeters (mm), and each experiment was performed in triplicate.

#### 2.5.2 Determination of the minimum inhibitory concentration (MIC) and minimum bactericidal concentration (MBC) of microcapsules against *L. innocua*

The MIC of microcapsules against *L. innocua* was determined in TSB using the broth microdilution method in 96-well microplates (Chakansin et al., 2022; Elshikh et al., 2016). A certain amount of UV-sterilized microcapsule powder was weighed, and dissolved in sterile TSB to prepare a microcapsule solution with an initial concentration of 80 mg/mL. Two-fold serial dilutions were then performed in the microtiter plate using TSB to obtain 100 μL of microcapsule solution in each well with final concentrations ranging from 0.078 to 80 mg/mL. After that, 100 μL of *L. innocua* culture at a concentration of 10^6^ CFU/mL in the exponential growth phase was added to each well and mixed well. Sterile TSB served as the blank control, and TSB inoculated with bacteria without microcapsules served as the negative control. The plates were then incubated at 37 °C for 24 h. After incubation, 30 μL of 0.015% (w/v) resazurin aqueous solution was added to each well and further incubated for 2-4 h for the observation of color change. The wells with no color change (the purple-blue resazurin color remained unchanged) were considered as above the MIC value. The MIC was defined as the lowest concentration of microcapsules in TSB that prevented visible bacterial growth after 24 h. The MBC was determined by subculturing 5 μL from wells above the MIC value directly onto the TSA plates, followed by incubation at 37 °C for 24 h. The MBC was defined as the lowest concentration at which no bacterial colonies were observed on the TSA plates. All experiments were performed in triplicate.

### 2.6 Antibiofilm activities of microcapsules against *L. innocua*

#### 2.6.1 Determination of the eradication efficiency of microcapsules against preformed *L. innocua* biofilms

The eradication efficiency of microcapsules against preformed *L. innocua* biofilms was evaluated using a crystal violet staining assay, according to previous studies with slight modification (Kwasny and Opperman, 2010; Müsken et al., 2010; Soulaimani et al., 2025). The assay was designed to evaluate the physical disruption and removal of preformed biofilm biomass rather than the viability of biofilm-associated cells. After fixation with 95% ethanol, the biofilms were exposed to the microcapsule formulations. Residual attached biomass was subsequently quantified by crystal violet staining. Therefore, the calculated biofilm eradication efficiency reflects biofilm matrix disintegration and biomass detachment from the polystyrene surface, rather than bactericidal activity against sessile cells. First, 200 μL of pre-cultured *L. innocua* suspensions at a concentration of 10^6^ CFU/mL in the exponential growth phase were inoculated into each well of sterile 96-well polystyrene microplates. The plates were then incubated at 37 °C for 24 h without shaking to form stable biofilms. The blank control was inoculated with 200 μL of TSB. After that, all bacterial suspensions and TSB were discarded from the plates, and each well was gently washed 1–2 times with sterile PBS buffer to remove residual planktonic bacteria cells. Then, 250 μL of 95% ethanol was added to each well to fix the formed biofilms for 30 min. The ethanol was poured out after fixation, and the plates were air-dried. Subsequently, 250 μL of microcapsule-TSB solution at different concentrations (MIC, MIC/2, MIC/4) was added to each well for biofilm elimination treatment, and the plates were further incubated at 37 °C for 0.5 h and 1 h. For the blank control (without biofilm) and negative control (with *L. innocua* biofilm), the same amount of TSB was added and incubated for the same duration. After treatment, all liquid in the plate was discarded, and each well was gently washed 1–2 times with PBS buffer and air-dried. Next, 0.1% crystal violet aqueous solution was added to each well for staining at room temperature in the dark for 45 min, and then washed with PBS 3-5 times to remove unbound dye. After air drying, 250 μL of 95% ethanol was added to dissolve the dye bound to the biofilm. After 10 min of treatment, the plate was placed in a microplate reader and shaken for 5 min, and the absorbance at 595 nm was measured. Each sample was repeated at least three times. The eradication efficiency of biofilm (EEB) was calculated according to the following formula:

(6) $$EEB\;\left(\%\right)=\frac{{OD}_{negative}-{OD}_{microcapsule}}{{OD}_{negative}-{OD}_{blank}}\times 100$$

where OD_negative_ is the optical density of formed *L innocua* biofilm treated with TSB after staining; OD_microcapsule_ is the optical density of formed *L innocua* biofilm treated with different microcapsules after staining; OD_blank_ is the optical density of TSB without biofilm after staining.

#### 2.6.2 Microscopic observation of *L. innocua* biofilm after microcapsule treatment

The formed biofilm treated with microcapsules was stained with crystal violet, following the same procedure as described above. The biofilm in the centre of each well of the 96-well microplate was then recorded using an EVOS M5000 imaging microscope at a magnification of ×10 (EVOS M5000, Thermo-Invitrogen, USA).

### 2.7 Biological activities of microcapsules after storage at 4 °C for one year

Due to the potential uncontrolled release of the encapsulated EOs and the degradation or denaturation of enzymes during storage, the biological activity of microcapsules may be affected over time. To assess the stability and functionality of the microcapsules, the prepared formulations were stored at 4 °C for one year. After the storage period, the antimicrobial and anti-biofilm activities of encapsulated carvacrol against *L. innocua* and its biofilm were determined using the same methodologies as previously described.

### 2.8 Statistical analysis

All experiments were carried out at least in triplicate. The results are reported as the mean and standard deviation of these measurements. Statistical analysis was performed by one-way ANOVA using SPSS software (IBM, version 26.0, USA). The significant differences between samples were determined by Tukey's honest significant difference (HSD) test with a confidence level of 95%.

## 3 Results and discussion

### 3.1 Particle size distribution and ζ-potential of the O/W nanoemulsions

The physicochemical characterization of pea protein-stabilized O/W nanoemulsions following the incorporation of pepsin or trypsin at pH 3.5, 7.0, and 10.0 was conducted (Table 2). At neutral and alkaline pH, the effect of pepsin was negligible due to its inactivation, while trypsin significantly improved stability. Dynamic light scattering (DLS) and ζ-potential analysis confirmed that the incorporation of trypsin notably increased the surface charge (−29.20 mV to −34.53 mV at pH 7.0; −32.92 mV to −34.85 mV at pH 10.0), and reduced the droplet size (284.60 nm to 232.52 nm at pH 7.0; 149.13 nm to 140.38 nm at pH 10.0), indicating a nanoscale dispersion with enhanced colloidal stability and resistance to aggregation. However, under acidic conditions (pH 3.5), the emulsion exhibited larger droplet sizes, higher PDI values, and lower absolute ζ-potential values. The incorporation of either enzyme led to destabilization of emulsions. This was particularly pronounced with pepsin, which resulted in a micron-scale droplet size (≈1.2 μm), a PDI value approaching 1.0, and a near-neutral ζ-potential. As explored in this study, severe emulsion instability was driven by pepsin's strong enzymatic activity and interfacial erosion at acidic pH, whereas the moderate interfacial hydrolysis of trypsin at neutral and alkaline pH improved interfacial packing and droplet refinement. These findings contribute to understanding the impact of nanoemulsion stability on the performance of microencapsulation.

**Table 2:** Effect of protease incorporation and formulation pH on the mean droplet size, polydispersity index (PDI), and ζ-potential of pea protein–stabilized carvacrol nanoemulsions.

Pea protein-based O/W nanoemulsions		Mean particle size (nm)	PDI	ζ-potential (mV)
pH 3.5 pea protein emulsion	Without enzyme	436.53 ± 29.72c	0.51 ± 0.07b	23.00 ± 1.06a
	With pepsin	1219.00 ± 146.16a	0.97 ± 0.05a	−1.43 ± 5.33c
	With trypsin	669.45 ± 130.54b	0.77 ± 0.23a	15.93 ± 3.93b
pH 7.0 pea protein emulsion	Without enzyme	284.60 ± 8.64cd	0.24 ± 0.01c	−29.20 ± 1.89d
	With pepsin	281.57 ± 3.76cd	0.26 ± 0.01c	−30.63 ± 1.32d
	With trypsin	232.52 ± 7.55d	0.24 ± 0.05c	−34.53 ± 0.92d
pH 10.0 pea protein emulsion	Without enzyme	149.13 ± 3.20d	0.26 ± 0.01c	−32.92 ± 0.94d
	With pepsin	183.72 ± 12.14d	0.38 ± 0.05bc	−32.64 ± 1.04d
	With trypsin	140.38 ± 5.79d	0.29 ± 0.02bc	−34.85 ± 1.05d

Note: Data are expressed as mean ± standard deviation (n = 3). Different superscript letters within the same column indicate significant differences among formulations (p < 0.05, one-way ANOVA followed by Tukey's HSD test).

### 3.2 Physicochemical characterization of the microcapsules

This section focused on preparation and characterization of carvacrol microcapsules based on the nine types of pea-protein-based nanoemulsions described above, with particular attention to the relationship between the stability of protease-incorporated emulsions and the performance of maltodextrin microcapsules. Maltodextrin was selected as the wall material because its low viscosity and neutral to slightly negative charge minimize interference with charged emulsions. The effects of three factors on encapsulation performance were also investigated: enzyme type (trypsin and pepsin), pH value (3.5, 7.0, and 10.0), and drying technique (spray-drying and freeze-drying). Proper control of these factors is critical for the retention of carvacrol and enzymes during microencapsulation and for preserving their biological activity.

#### 3.2.1 Moisture content and water activity (Aw)

The moisture content of microcapsule powders has a significant impact on their long-term stability and shelf life. Excessive moisture can promote oxidation and hydrolysis of encapsulated components, facilitate microbial growth, and directly affect the structural properties of the microcapsules. The moisture content of spray-drying microcapsules ranged from 4.03% to 7.70%, whereas those produced by freeze drying ranged from 2.70% to 6.88% (Table 3). No significant differences were observed between the two drying techniques, and most microcapsules had a moisture content that met the requirements for long-term storage (<6%) (Bajac et al., 2022). Furthermore, there was no significant difference in moisture content among microcapsules prepared under different pH conditions, consistent with the report by Benkirane et al. (2024). However, the presence of enzymes under acidic conditions markedly increased the moisture content of spray-drying microcapsules containing pepsin and trypsin (SP3.5 and ST3.5), and freeze-drying microcapsules containing trypsin (FT3.5) to 7.70%, 6.52%, and 6.16%, respectively. Under neutral conditions, trypsin incorporation increased the moisture content of freeze-drying microcapsules (FT7) to 6.88%. These elevated moisture levels may pose risks of bacterial contamination and lipid oxidation during long-term storage. This effect could be attributed to emulsion destabilization caused by enzyme treatment under acidic conditions. Flocculated or coalesced emulsions tend to exhibit abnormal encapsulation behavior during drying, including uneven wall-material distribution and reduced efficiency. Under neutral conditions, the high activity of trypsin may hydrolyze pea proteins, generating more small peptides with enhanced hygroscopicity. In addition, the effects of enzyme type and pH on the A_w_ of microcapsules were consistent with the moisture content results. However, all freeze-dried microcapsules showed significantly lower A_w_ values (0.087–0.188) than their spray-dried counterparts (0.194–0.296) (Table 3), and all values were clearly below the critical threshold of 0.3 for microbial growth and lipid oxidation (da Silva Júnior et al., 2023). This difference may be attributed to the porous structures generated during freeze-drying, which enable more thorough removal of free water, whereas spray-drying tends to produce denser structures (Muhoza et al., 2023).

**Table 3:** Effects of protease incorporation, formulation pH, and drying method on the physicochemical and particle-size properties of pea protein–based carvacrol microcapsules.

Microcapsules	Moisture content (%)	Water activity (A_w_)	Hygroscopicity (%)	Particle size, D_[4,3]_ (μm)	Particle size, D_[3,2]_ (μm)	Span
SN7	5.25 ± 1.15bc	0.217 ± 0.008c	18.85 ± 1.38a	12.30 ± 2.72cd	6.12 ± 0.35d	1.85 ± 0.36c
SP7	5.94 ± 0.35 abc	0.219 ± 0.014c	17.81 ± 0.43a	28.93 ± 3.32a	6.19 ± 0.23d	9.21 ± 2.30a
ST7	4.99 ± 0.61bc	0.209 ± 0.015c	19.61 ± 2.13a	9.11 ± 1.82cde	5.14 ± 0.21e	1.76 ± 0.13c
SN3.5	5.27 ± 0.43bc	0.235 ± 0.012bc	17.77 ± 0.66a	8.60 ± 1.39de	5.36 ± 0.21e	1.70 ± 0.07c
SP3.5	7.70 ± 0.56a	0.296 ± 0.015a	18.07 ± 0.81a	23.50 ± 1.57b	7.06 ± 0.11c	4.65 ± 0.60b
ST3.5	6.52 ± 0.58 ab	0.281 ± 0.002 ab	16.84 ± 0.45a	7.71 ± 0.79e	5.25 ± 0.16e	1.63 ± 0.03c
SN10	4.03 ± 0.11c	0.194 ± 0.007c	18.08 ± 0.55a	12.80 ± 0.07c	8.83 ± 0.10a	1.59 ± 0.02c
SP10	4.41 ± 0.26c	0.230 ± 0.009bc	17.76 ± 0.73a	12.30 ± 0.05cd	8.06 ± 0.02b	1.74 ± 0.00c
ST10	4.49 ± 0.15c	0.221 ± 0.006c	16.85 ± 0.64a	11.80 ± 0.04cd	7.83 ± 0.04b	1.70 ± 0.01c
FN7	5.50 ± 1.10 ab	0.183 ± 0.004a	17.25 ± 0.45a	266.67 ± 16.98b	87.37 ± 7.01c	2.59 ± 0.07bc
FP7	5.05 ± 1.30 ab	0.174 ± 0.004a	17.11 ± 2.74a	253.80 ± 6.76b	105.40 ± 2.94b	1.90 ± 0.01e
FT7	6.88 ± 1.90a	0.188 ± 0.010a	16.87 ± 2.48a	253.00 ± 4.00b	102.40 ± 1.50b	1.87 ± 0.01e
FN3.5	5.75 ± 0.22 ab	0.172 ± 0.001a	18.44 ± 1.86a	196.80 ± 8.45c	62.50 ± 1.58e	2.37 ± 0.10cd
FP3.5	5.55 ± 1.47 ab	0.173 ± 0.005a	19.54 ± 0.93a	330.20 ± 12.51a	122.80 ± 2.93a	2.71 ± 0.04 ab
FT3.5	6.16 ± 1.06 ab	0.167 ± 0.003a	17.48 ± 0.27a	312.80 ± 25.74a	99.58 ± 5.37b	2.45 ± 0.07bcd
FN10	3.44 ± 0.20 ab	0.087 ± 0.002c	17.57 ± 0.32a	172.40 ± 4.41c	70.56 ± 1.60d	2.23 ± 0.06d
FP10	2.70 ± 0.11b	0.113 ± 0.003b	16.18 ± 2.06a	252.00 ± 30.11b	83.76 ± 4.68c	2.66 ± 0.16b
FT10	3.30 ± 0.28 ab	0.093 ± 0.003bc	17.46 ± 0.48a	158.40 ± 15.78c	55.78 ± 1.95e	2.95 ± 0.27a

Note: Data are expressed as mean ± standard deviation (n = 3). Different superscript letters within the same column indicate significant differences among formulations (p < 0.05, one-way ANOVA followed by Tukey's HSD test).

#### 3.2.2 Hygroscopicity

Hygroscopicity refers to the ability of microcapsules to absorb moisture from the storage environment, and high hygroscopicity can lead to structural degradation and loss of activity of the encapsulated components. After storage at 76% relative humidity for 7 days, the hygroscopicity of all microcapsules ranged from 16.18% to 19.61%, and no significant effects of drying technique, pH, or enzyme incorporation on hygroscopicity were observed (Table 3). These findings are consistent with those reported by Benkirane et al. (2024), fall within the acceptable range (<20%) for storage, and are notably superior to pectin–alginate-based microcapsules encapsulating carvacrol, which exhibited a hygroscopicity of 34.44 g water/100 g powder (Sun et al., 2019).

#### 3.2.3 Particle size of microcapsules

**Fig. 1:**
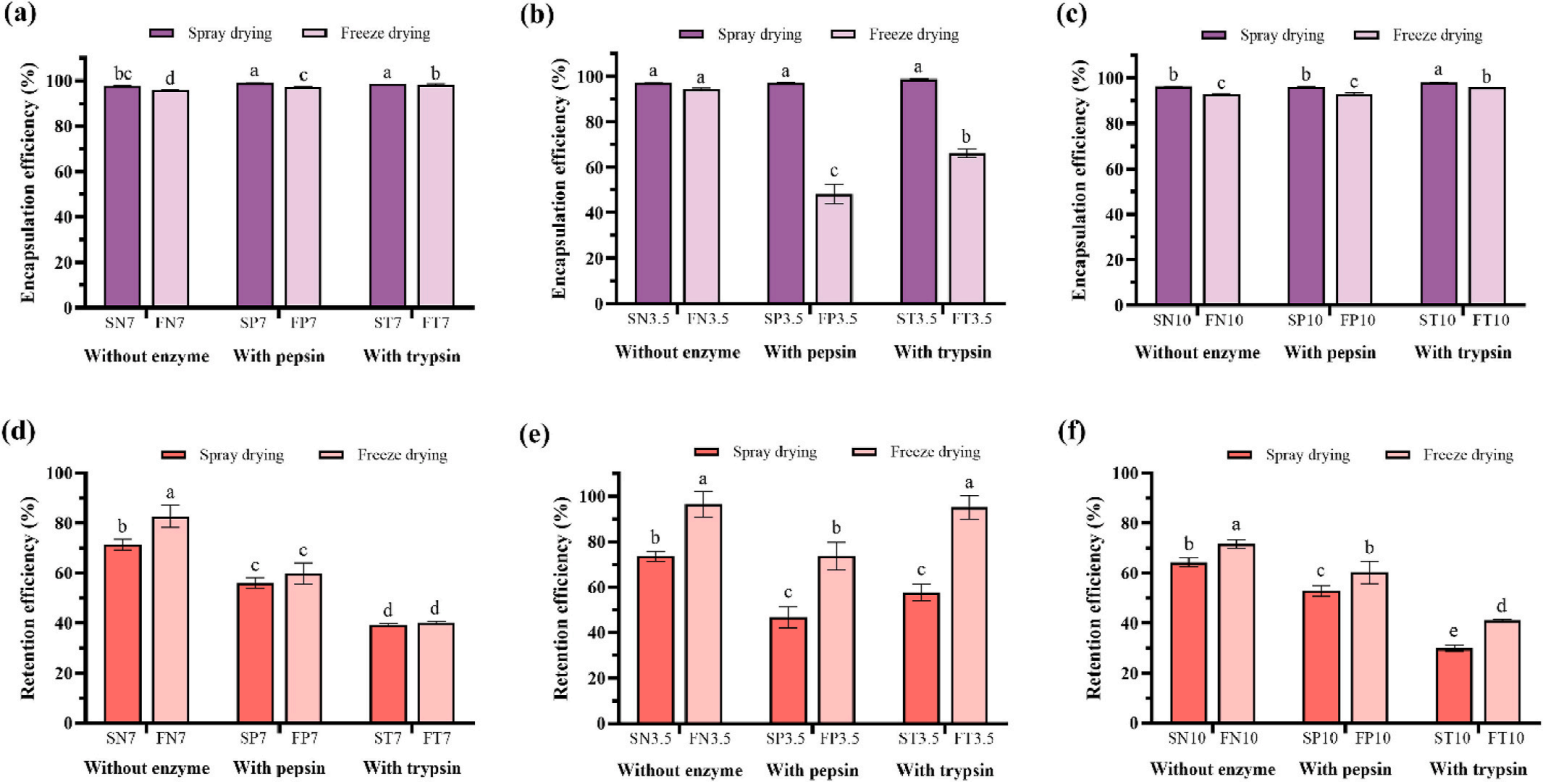
Encapsulation efficiency and retention efficiency of carvacrol within enzyme-free and enzyme-containing microcapsules at pH 7.0, pH 3.5 and pH 10.0 under spray-drying and freeze-drying. (a-c). Encapsulation efficiency of carvacrol microcapsules at pH 7.0, pH 3.5 and pH 10.0; (d-f). Retention efficiency of carvacrol microcapsules at pH 7.0, pH 3.5 and pH 10.0. Different letters indicate statistically significant differences between samples.

**Fig. 2:**
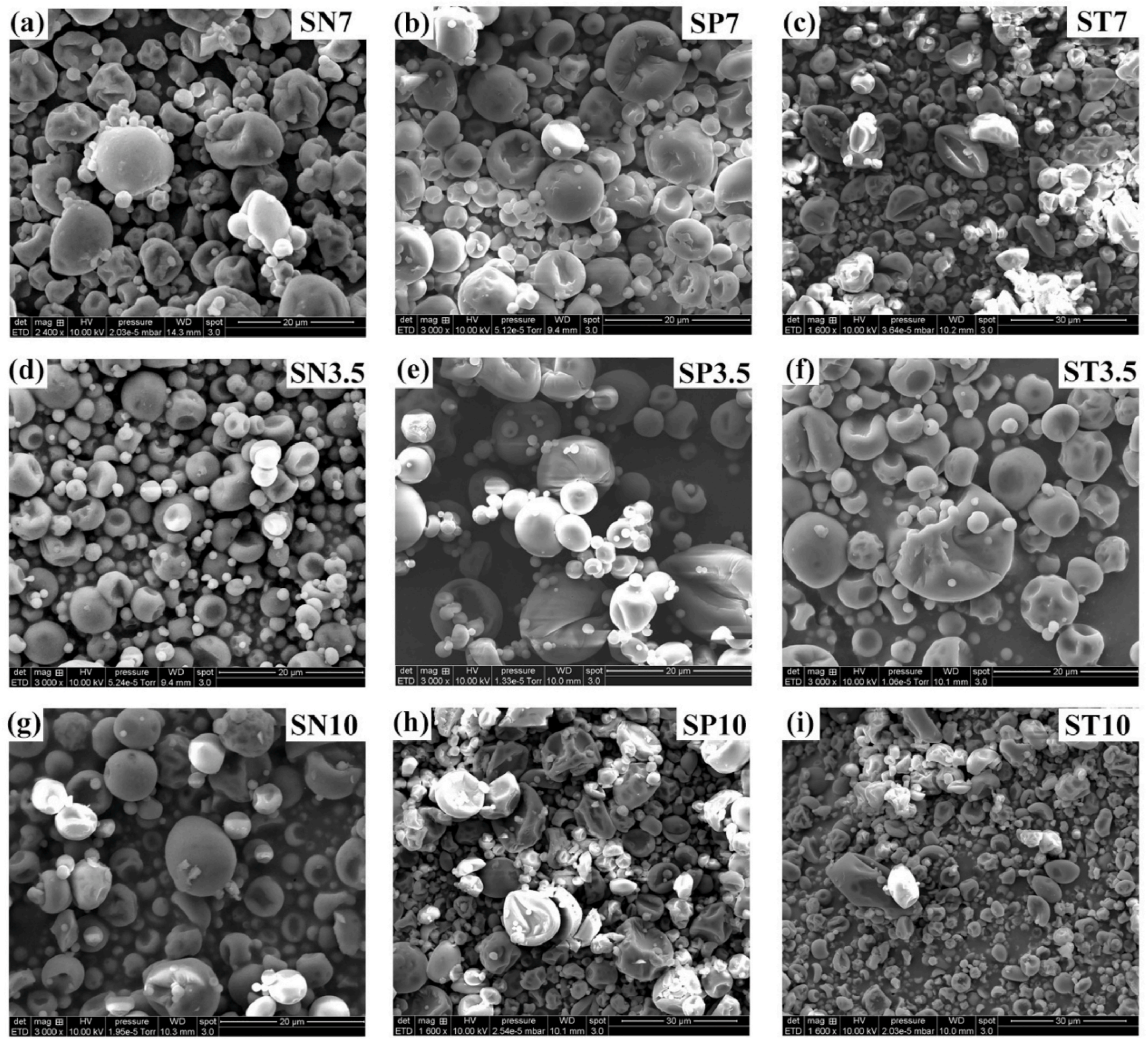
Scanning electron microscopy (SEM) images of carvacrol microcapsules with different formulations obtained by spray-drying. Scale bars: 20 or 30 μm; magnifications:×1600, ×2400, or ×3000, as indicated in each panel.

The particle size distribution parameters of all microcapsule formulations, including D_4,3_, D_3,2_, and Span, are summarized in Table 3, where the effects of pH, drying technique, and enzyme incorporation were examined. D_4,3_ is more sensitive to the presence of large particles within the capsules, whereas D_3,2_ reflects the contribution of smaller ones, concurrent reporting of both parameters offers a more robust characterization of particle size distribution. The statistical analysis revealed that the drying technique exerted the strongest influence on microcapsule size. Spray-dried powders exhibited markedly smaller particle sizes (D_4,3_ from 7.71 to 28.93 μm, D_3,2_ from 5.14 to 8.83 μm) compared with their freeze-dried counterparts (D_4,3_ from 158.4 to 330.2 μm, D_3,2_ from 55.78 to 122.8 μm), consistent with reports by Baghi et al. (2023). This pronounced discrepancy arises from the atomization of emulsions during spray-drying, where fine droplets undergo rapid solidification and limited coalescence, yielding small and predominantly spherical particles. In contrast, freeze-drying forms porous and irregular sponge-like matrices that require subsequent milling or grinding, making it difficult to obtain finer powders. These morphological distinctions are further corroborated by the SEM images (Fig. 1, Fig. 2). The lyophilized powders also displayed larger gaps between D_4,3_ and D_3,2_, together with higher Span values (1.87–2.95), reflecting the heterogeneous nature of particle breakage during milling.

Emulsion pH exerted a significant impact on the particle size of freeze-dried microcapsules, particularly in enzyme-incorporated formulations. The largest lyophilized particles were formed under acidic conditions (pH 3.5), followed by pH 7, whereas alkaline conditions (pH 10) resulted in the smallest particles, possibly due to pH-dependent changes in nanoemulsion stability and interfacial organization. The influence of pH was less evident in spray-dried samples; however, SP7 exhibited larger particle size and Span, suggesting reduced colloidal stability. Enzyme incorporation further modulated the particle size distribution. Across both drying methods, pepsin-incorporated microcapsules consistently produced larger particles, particularly at pH 3.5 and 7, suggesting that active pepsin disrupted the structural integrity of the wall matrix and promoted droplet or particle aggregation. By comparison, trypsin exerted a milder effect: particle sizes remained stable in spray-dried powders, and at pH 10, trypsin-incorporated freeze-dried powders showed the smallest particle sizes, attributed to the improved stability and reduced droplet size of the emulsion under alkaline conditions (as shown in Table 2). These findings demonstrate that the microcapsule particle size distribution was governed primarily by the drying techniques, with secondary contributions from enzyme incorporation and emulsion pH. And pepsin consistently promoted particle enlargement across conditions, whereas trypsin induced modest effects.

#### 3.2.4 Encapsulation characteristics

**Fig. 3:**
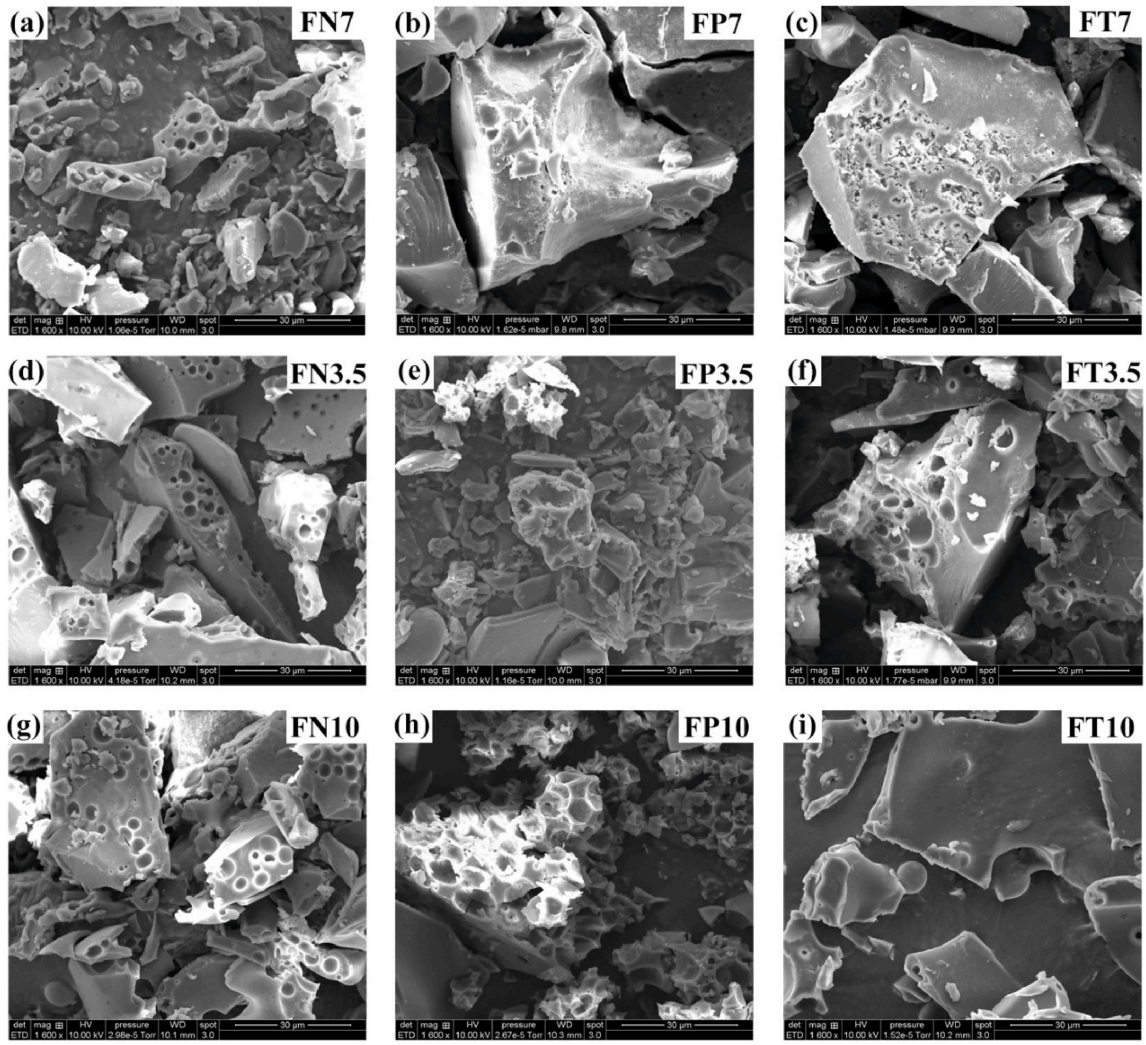
Scanning electron microscopy (SEM) images of carvacrol microcapsules with different formulations obtained by freeze-drying. Scale bar: 30 μm; magnification: ×1600.

The encapsulation behavior of the microcapsules was strongly affected by the drying technique, formulation pH, and protease treatment. As shown in Table 4, freeze-drying yielded substantially higher microcapsule recovery (75.29–85.29%) than spray-drying (61.88–72.33%). Spray-dried powders consistently exhibited lower surface oil contents and essential-oil retention efficiency (RE), alongside markedly higher encapsulation efficiency (EE > 96-99%). In contrast, freeze-dried powders possessed higher loading capacity (LC) yet displayed elevated surface oil levels and reduced EE, particularly at pH 3.5, owing to the formation of porous and fragile matrices (Fig. 3).

**Table 4:** Effects of protease incorporation, formulation pH, and drying method on the process yield and carvacrol encapsulation characteristics of pea protein–based microcapsules.

Drying technique	Microcapsules	Process yield (%)	Surface oil content (mg/g)	Loading capacity (mg/g)
Spray drying	SN7	68.06	3.00 ± 0.15b	137.26 ± 3.62b
	SP7	61.88	0.96 ± 0.10c	107.44 ± 3.32c
	ST7	64.54	0.95 ± 0.31c	75.16 ± 0.91d
	SN3.5	71.98	3.86 ± 0.25b	141.42 ± 3.21b
	SP3.5	66.72	2.47 ± 0.40b	89.49 ± 7.76c
	ST3.5	67.49	1.24 ± 0.22b	110.24 ± 5.98c
	SN10	72.33	4.49 ± 1.30c	123.53 ± 2.93b
	SP10	69.67	3.75 ± 0.76c	99.85 ± 4.60c
	ST10	66.38	1.00 ± 0.56d	57.44 ± 2.03e
Freeze drying	FN7	85.29	9.47 ± 1.09a	238.29 ± 11.57a
	FP7	80.00	2.94 ± 0.16b	114.84 ± 6.86c
	FT7	75.29	1.39 ± 0.34bc	76.92 ± 0.50d
	FN3.5	81.40	9.85 ± 0.85b	185.43 ± 9.44a
	FP3.5	83.37	69.51 ± 6.99a	141.10 ± 10.11b
	FT3.5	85.19	60.74 ± 2.08a	182.22 ± 8.68a
	FN10	82.77	9.67 ± 1.05a	137.67 ± 2.86a
	FP10	82.05	8.22 ± 1.02b	115.62 ± 7.51b
	FT10	80.56	3.10 ± 1.15c	78.62 ± 0.66d

Note: Data are expressed as mean ± standard deviation (n = 3). Different superscript letters within the same column indicate significant differences among formulations (p < 0.05, one-way ANOVA followed by Tukey's HSD test).

*Effect of drying technique.* Spray-dried microcapsules showed low surface oil contents (0.95–4.49 mg/g) and maintained high EE (96.24–99.11%) across all pH conditions (Table 4, Fig. 1), reflecting the formation of dense, spherical particles that effectively entrapped the oil core, consistent with the 85.89% EE reported for carvacrol microcapsules encapsulated with sodium caseinate by Brahmi et al. (2025b). The rapid droplet drying and limited porosity of these particles likely facilitated the efficient encapsulation of carvacrol within the particle interior while minimizing its exposure at the surface. However, the generally lower LC (57.44–141.42 mg/g) and RE values (29.98–73.54%) suggested thermal evaporation or volatilization losses during hot-air drying. In contrast, freeze-dried samples exhibited significantly higher LC and RE (76.92–238.29 mg/g; 40.15–96.43%), indicating minimal core-oil loss during low-temperature dehydration. Nevertheless, their surface oil contents were markedly increased, especially in enzyme-treated samples at pH 3.5 (FP3.5 and FT3.5 > 60 mg/g, Table 4). Although EE remained relatively high (>90%), it was significantly lower than that of spray-dried powders, particularly at pH 3.5, in agreement with Baghi's reports regarding lyophilized microcapsules (Baghi et al., 2023). These observations suggest that the porous, brittle matrices formed during freeze-drying restrict the encapsulation of surface carvacrol and promote outward carvacrol migration, thereby compromising overall encapsulation stability. Besides, the differences between the two drying methods were governed not only by the final particle morphology but also by their distinct water migration and mass transfer mechanisms. During spray-drying, rapid evaporation generates a strong outward moisture flux and promotes early formation of a relatively dense surface layer. This rapid shell formation can restrict subsequent diffusion of carvacrol toward the particle surface, thereby contributing to low surface oil content and high EE. However, atomization greatly increases the gas–liquid interfacial area, while the elevated drying temperature and rapid mass transfer may promote volatilization of carvacrol, consequently reducing its overall LC and RE (Phanse and Chandra, 2024; Laina et al., 2024). By contrast, freeze-drying removes water through ice sublimation under low-temperature and vacuum conditions, thereby limiting heat-induced loss of volatile compounds and favoring higher carvacrol loading and retention. During freezing, however, ice-crystal growth concentrates proteins, maltodextrin, and oil within the unfrozen phase. Subsequent sublimation leaves interconnected pores and mass-transfer channels that can facilitate carvacrol redistribution and migration toward the powder surface, particularly when the initial emulsion is destabilized by proteolysis (Napiórkowska et al., 2023; Laina et al., 2024). Consequently, freeze-dried powders may retain more total carvacrol while simultaneously exhibiting higher surface oil content and lower EE.

*Effect of pH*. The influence of pH on encapsulation behavior was most pronounced in freeze-dried samples. At pH 3.5, EE declined sharply in the presence of pepsin or trypsin (FP3.5 = 48.18%; FT3.5 = 66.33%), accompanied by extremely high surface carvacrol levels (69.51 and 60.74 mg/g), indicating severe emulsion destabilization, and pronounced phase separation prior to freezing. Spray-dried powders were less sensitive to pH, maintaining high EE (≥95%) under all conditions, although minor reductions in RE were observed, likely due to weakened interfacial strength during atomization. Additionally, at pH 7.0 and 10.0, all microcapsules displayed reduced surface oil contents, especially after enzyme incorporation, with spray-dried powders showing the lowest values. This suggests enhanced colloidal stability and stronger interfacial membranes under neutral and alkaline conditions, which provide a more favorable environment for carvacrol encapsulation. The observed pH-dependent encapsulation behavior is consistent with previous reports on pea-protein-based delivery systems. Baghi et al. (2023) showed that PPI-stabilized trans-cinnamaldehyde formulations prepared at pH 3.0 and 7.0 exhibited different emulsion and powder properties, confirming that the ionization and interfacial behavior of PPI strongly depend on formulation pH. Similarly, Wang et al. (2022a) demonstrated that pH governed the organization of PPI–sugar beet pectin interfacial structures and consequently affected the architecture and encapsulation properties of the resulting oil microcapsules. It should be noted that electrostatic complexation with an oppositely charged polysaccharide may favor compact structures under acidic conditions in coacervate systems. In the present PPI–maltodextrin system, however, the absence of such interfacial complexation, together with reduced PPI solubility and pepsin-induced destabilization at pH 3.5, resulted in higher surface carvacrol and lower EE. In contrast, the stronger surface charge and improved emulsion stability at pH 7.0 and 10.0 favored more uniform wall-material deposition and reduced carvacrol migration.

*Effect of enzyme incorporation*. Protease incorporation produced notable effects on the encapsulation performance of microcapsules. Enzyme-incorporation samples consistently exhibited reduced LC and RE (Table 4, Fig. 1), possibly because trypsin and pepsin exhibited hydrolytic activity against pea protein under their optimal pH conditions, thereby weakening the interfacial membrane and facilitating carvacrol permeation or volatilization, particularly under the high temperatures of spray-drying. Nevertheless, even when total carvacrol content decreased, trypsin treatment at pH 7.0 and 10.0 promoted the formation of a more densely packed interfacial protein network due to controlled proteolysis. This enhanced interfacial structure impeded carvacrol redistribution, reduced its surface migration, lowered measured surface oil contents, and ultimately improved EE, consistent with the smaller droplet sizes and enhanced emulsion stability observed under neutral and alkaline conditions (Table 2). At pH 3.5, however, enzyme-treated freeze-dried microcapsules exhibited dramatically elevated surface oil and reduced EE, indicating the severe disruption of emulsion integrity before drying, in line with earlier observations. Recent studies further indicate that the influence of enzymatic modification on encapsulation performance depends strongly on the extent of protein hydrolysis. Gomes and Kurozawa (2024) demonstrated that rice protein hydrolysates with different degrees of hydrolysis exhibited distinct interfacial adsorption and film-forming behaviors during the microencapsulation of orange essential oil, indicating that controlled hydrolysis can improve the mobility and organization of protein fragments at the oil–water interface. Conversely, Beigmohammadi et al. (2024) reported that enzymatically hydrolyzed whey protein produced smaller droplets but generated broken and irregular essential-oil microparticles, with EE values of only 58.2–61.3%, whereas formulations retaining better wall integrity reached an EE of approximately 89%. These observations support the present results: moderate trypsin-mediated remodeling at pH 7.0 and 10.0 favored interfacial rearrangement and limited surface migration of carvacrol, whereas extensive pepsin-mediated hydrolysis at pH 3.5 weakened the interfacial layer and substantially reduced encapsulation performance, particularly in FP3.5, which exhibited an EE of 48.18% and a surface carvacrol content of 69.51 mg/g.

Overall, spray-drying microcapsules exhibited superior interfacial encapsulation performance, characterized by low surface carvacrol and high EE, whereas freeze-drying, despite yielding higher LC and RE, facilitated carvacrol migration and structural fragility, making it less favorable for long-term storage. Under both techniques, pepsin substantially diminished encapsulation performance, particularly under acidic conditions, whereas trypsin exerted relatively milder effects. These findings highlight the intricate interplay among pH, enzyme incorporation, and drying technique in governing microcapsule EE and overall encapsulation behavior.

#### 3.2.5 Morphological characterization of microcapsules by SEM

The SEM observations of spray-dried and freeze-dried microcapsules are presented in Fig. 2, Fig. 3, respectively. Most spray-dried microcapsules exhibited uniform and well-defined spherical morphologies, with occasional concavities and surface cracks (Fig. 2). At pH 3.5, enzyme incorporation led to pronounced particle aggregation and size enlargement, particularly in the presence of pepsin (SP3.5), consistent with the reported particle-size data. However, freeze-dried microcapsules displayed irregular, glass-like fractured structures with evident surface pores and cavities, and their morphology remained largely unaffected by formulation differences, which aligns with the findings of Benkirane et al. (2024). The porous and fragile appearance is attributed to ice-crystal formation and subsequent sublimation during freeze-drying. The morphological differences were also consistent with the quantitative particle-size and encapsulation results. Spray-dried powders exhibited D_4,3_ values of 7.71–28.93 μm and formed relatively dense particles with limited visible cracking, whereas freeze-dried powders showed much larger D_4,3_ values of 158.4–330.2 μm and fractured porous matrices. These pores and cavities may provide interconnected pathways for the outward migration and surface exposure of carvacrol. Similar structural differences between spray-dried and freeze-dried EO microcapsules have been widely reported in recent studies. For example, recent investigations on food-grade encapsulation systems have shown that spray drying generally produces smaller, more compact particles with lower surface oil contents and higher EE, whereas freeze drying tends to generate larger, highly porous matrices that facilitate mass transfer but may increase the exposure of encapsulated bioactive compounds to environmental factors (Čulina et al., 2023; Enascuta et al., 2025). Likewise, studies on protein- and polysaccharide-based delivery systems have demonstrated that porous structures formed during sublimation can enhance the accessibility of encapsulated compounds after rehydration, while dense spray-dried matrices are more effective in limiting premature migration and improving storage stability (Asres et al., 2025b). The morphological characteristics observed in the present study therefore agree well with the general trends reported for EO microencapsulation systems. However, because pore dimensions and shell thickness were not directly measured, the SEM observations should be interpreted together with particle size, surface carvacrol content, and EE rather than as a quantitative assessment of internal porosity.

#### 3.2.6 ATR-FTIR spectroscopy

**Fig. 4:**
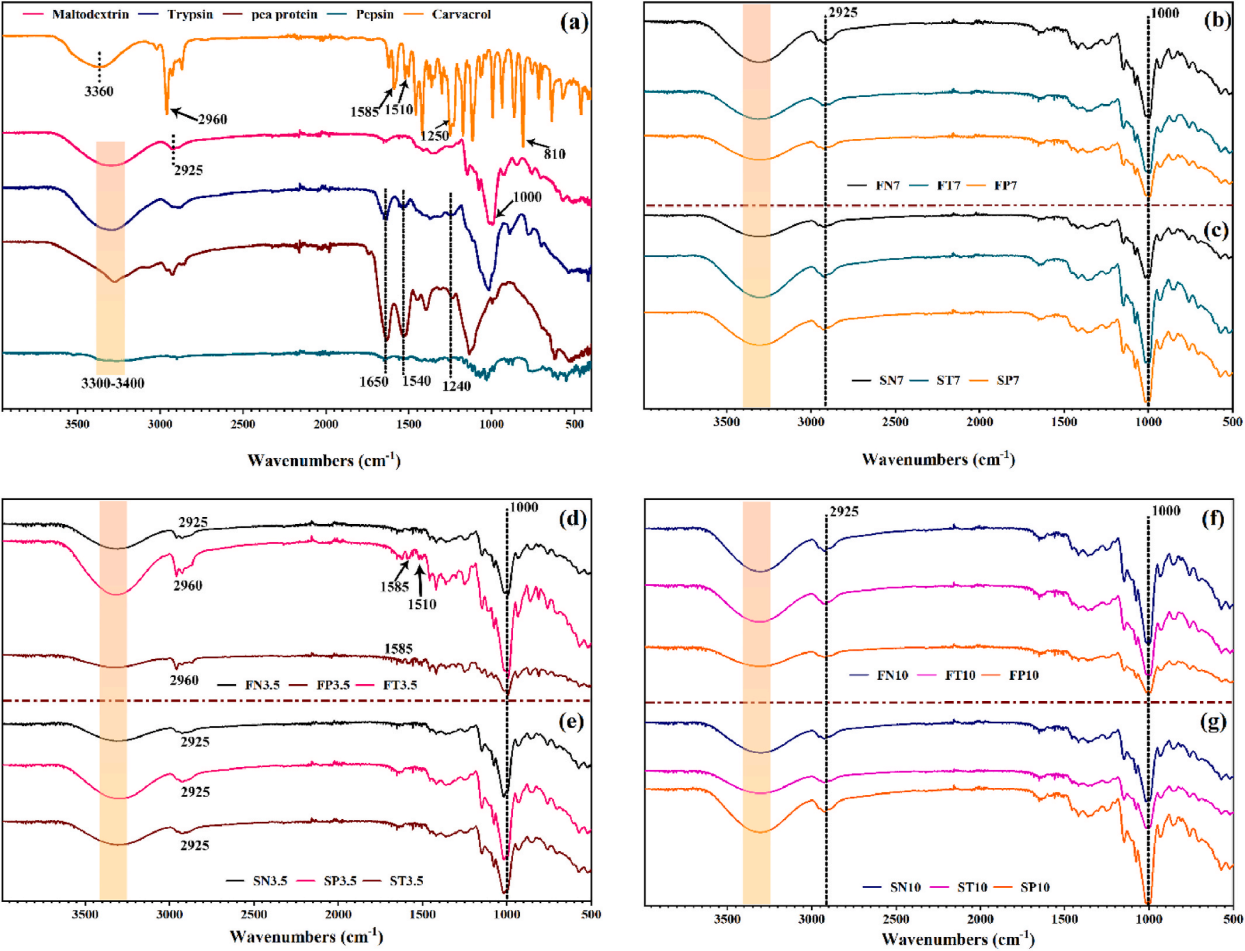
ATR-FTIR spectra of the individual formulation components and carvacrol-loaded microcapsules. (a) Reference spectra of maltodextrin, trypsin, pea protein isolate, pepsin, and carvacrol; (b, d, and f) freeze-dried microcapsules prepared at pH 7.0, 3.5, and 10.0, respectively; and (c, e, and g) spray-dried microcapsules prepared at pH 7.0, 3.5, and 10.0, respectively. The main characteristic absorption bands are marked in the spectra. All spectra were recorded over the range of 4000–400 cm^−1^ and are presented over the range of 4000–500 cm^−1^ for clarity.

The FTIR spectra provided molecular-level insights into the interactions among pea protein, maltodextrin, carvacrol, and proteases, as well as the structural alterations occurring during enzyme treatment and subsequent spray-drying or freeze-drying (Fig. 4). Fig. 4a illustrated the FTIR spectra of the raw materials used for microcapsule fabrication. Pea protein displayed the characteristic proteinaceous absorption bands, including the amide I (around 1650 cm^−1^) and amide II (about 1540 cm^−1^) bands, corresponding to C=O stretching and N–H bending vibrations, respectively, as well as an amide III band near 1240 cm^−1^, consistent with previous reports by Sun et al. (2022) and Xia et al. (2024). Trypsin and pepsin exhibited similar spectral features, though variations in the relative intensities of the amide regions reflect differences in their secondary structural compositions. Maltodextrin showed a broad O–H stretching band at 3300-3400 cm^−1^ and strong C–O–C/C–O stretching bands in the 1150-950 cm^−1^ region, typical of polysaccharides and in agreement with the findings of Costa et al. (2015). The spectra of all microcapsules (Fig. 4b–g) resembled that of maltodextrin, indicating acceptable encapsulation behavior. Carvacrol exhibited a broad phenolic O–H stretching band at approximately 3360 cm^−1^, aliphatic C–H stretching vibrations at around 2960 cm^−1^, and characteristic aromatic C=C stretching bands near 1585 and 1510 cm^−1^. The band at approximately 1250 cm^−1^ was assigned to phenolic C–O stretching, while the strong absorption in the 820–760 cm^−1^ region corresponded to out-of-plane aromatic C–H bending, in agreement with Brahmi et al. (2025a) and Charles et al. (2022).

As shown in Fig. 4b–g, the spectra of all microcapsules were dominated by the maltodextrin matrix, while the characteristic aromatic peaks of carvacrol were markedly attenuated or absent in most formulations. This substantial suppression of carvacrol signals indicated effective encapsulation of the oil within the wall matrix rather than exposed on the particle surface. Furthermore, since the amide signals of trypsin and pepsin overlapped with those of pea protein, no new diagnostic peaks emerged, only subtle variations in the amide I-II regions could be used to indirectly assess changes in protein secondary structure. More pronounced amide bands and a broader O–H/N–H stretching region were observed in microcapsules prepared at neutral and alkaline pH (Fig. 4b–c, f-g), indicative of enhanced protein solubility and a more cohesive protein-polysaccharide network.

In contrast, microcapsules formulated at pH 3.5 (Fig. 4d–e), particularly those containing pepsin or trypsin, showed substantial reductions in amide I and II band intensities, pronounced broadening around 3300 cm^−1^, and re-emergence of carvacrol's aromatic and aliphatic characteristic peaks, most notably in freeze-dried powders (Fig. 4d). These spectral alterations suggested extensive enzymatic hydrolysis of pea protein under acidic conditions, leading to severe disruption of the interfacial membrane and substantial migration of carvacrol toward the particle surface. Such structural deterioration corresponds well with the severe emulsion instability and high surface oil contents found in freeze-dried FP3.5 and FT3.5. Furthermore, spray-dried microcapsules displayed sharper amide bands and more pronounced maltodextrin signals (1150-950 cm^−1^), suggesting the formation of a denser and more cohesive matrix that favors effective encapsulation of carvacrol. This observation aligns with previous conclusions that spray-drying primarily induces physical dehydration without altering the chemical structure of core materials (Costa et al., 2015). Conversely, freeze-dried powders exhibited more prominent carvacrol characteristic peaks, in line with their more porous and fragile microstructures and the correspondingly higher level of surface oil exposure. These results demonstrate that neutral and alkaline pH conditions favor the development of a stable, hydrogen-bonded and protein-reinforced matrix, whereas acidic pH in the presence of proteases results in pronounced structural degradation, enhanced carvacrol diffusion to the particle surface, and diminished encapsulation performance.

#### 3.2.7 Mechanistic linkage between interfacial proteolysis and microcapsule formation

The contrasting effects of pepsin and trypsin on microcapsule formation can be interpreted based on the pH-dependent interfacial mechanisms established in our previous nanoemulsion study (Ji et al., 2025). Briefly, under neutral and alkaline conditions, trypsin induced limited remodeling of the PPI-stabilized interface, accompanied by smaller droplet sizes and increased negative surface charge. Such controlled proteolysis may increase the conformational flexibility and interfacial mobility of protein fragments, allowing them to rearrange more efficiently around carvacrol droplets and maintain stronger electrostatic repulsion. In contrast, under acidic conditions, active pepsin caused extensive degradation of the PPI interfacial layer, reduced electrostatic stabilization, and promoted droplet aggregation and phase separation.

More broadly, the conversion of protein-stabilized emulsions into dry particles should be considered a processing-history-dependent restructuring process. Previous studies on shear-processed gluten systems demonstrated that mechanical deformation can redistribute protein domains and promote protein-network development, while local shear intensity and processing time determine whether protein structures undergo formation, disruption, or reaggregation (Peighambardoust et al., 2004, 2007). Although these studies concerned wheat-gluten systems rather than PPI-stabilized emulsions, they support the general principle that the structural state of proteins before and during processing strongly influences the architecture of the final material. In the present system, the protease-conditioned interfacial states established before drying were therefore expected to respond differently to atomization, rapid dehydration, freezing, and sublimation, resulting in distinct microcapsule morphologies and encapsulation behaviors.

These distinct interfacial states were subsequently translated into different drying and encapsulation outcomes. The relatively stable trypsin-conditioned emulsions prepared at neutral and alkaline pH favored more uniform wall-material distribution during drying and limited the migration of carvacrol toward the particle surface. This behavior was consistent with the formation of denser spray-dried particles, low surface oil contents (0.95–1.00 mg/g), and high EE (>95%). By contrast, pepsin-induced destabilization at pH 3.5 produced heterogeneous feed emulsions before drying. The resulting freeze-dried powders exhibited porous and fragile structures, together with high surface oil content (69.51 mg/g) and markedly reduced EE (48.18%), indicating incomplete entrapment and enhanced outward migration of carvacrol.

These structural differences also provide a basis for interpreting the subsequent biological performance. Dense spray-dried particles may restrict immediate diffusion but protect carvacrol against premature loss, whereas porous freeze-dried matrices facilitate more rapid short-term release while providing weaker long-term protection. After rehydration, retained enzyme may additionally hydrolyze proteinaceous EPS of biofilm and enhanced carvacrol accessibility, thereby connecting the protease-conditioned interface with the improved antibiofilm performance of the resulting microcapsules.

### 3.3 Antibacterial activity of microcapsules against gram-positive and gram-negative bacteria

**Fig. 5:**
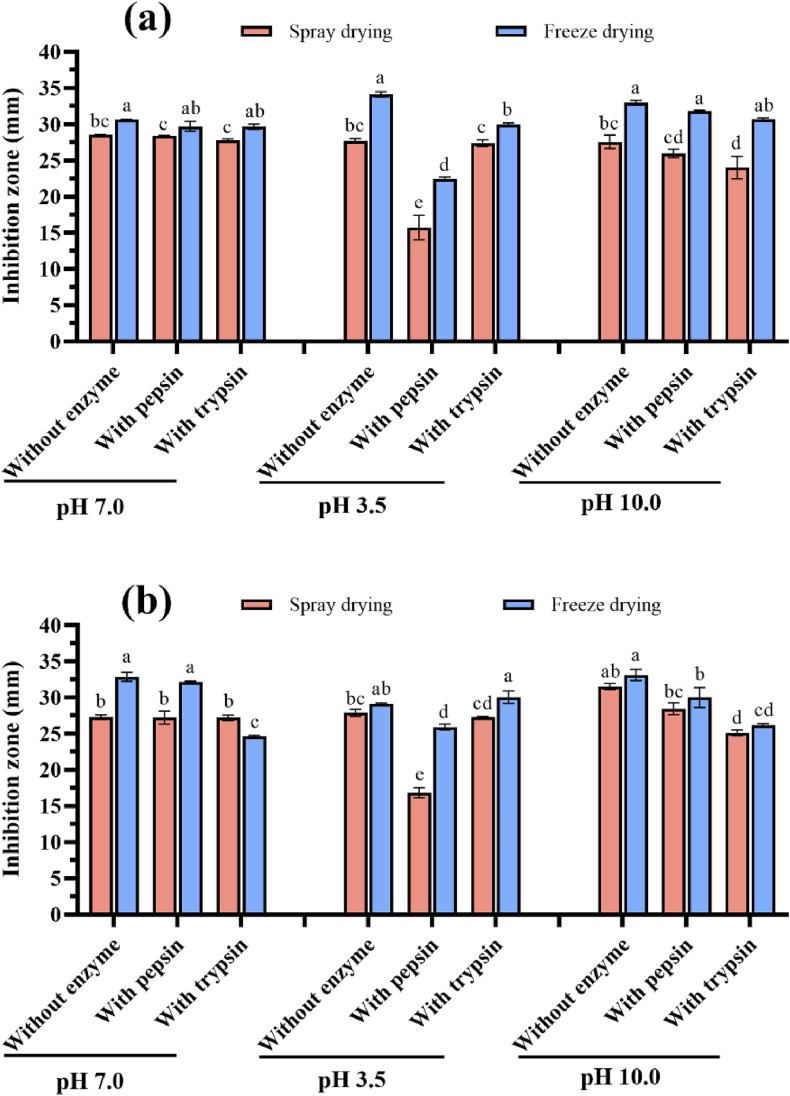
Antibacterial activity of pea protein–based microcapsules encapsulating carvacrol with/without enzyme incorporation against (a) *E. coli* and (b) *L. innocua*. Presentation of the inhibition zone diameter (mm). Different letters indicate statistically significant difference between samples at each pH condition.

The antibacterial efficacy of carvacrol could be influenced by variations in encapsulation formulations, including pH, protease incorporation, and drying technique. As shown in Fig. 5, the antimicrobial activities of pea protein-based carvacrol microcapsules against *E. coli* and *L. innocua* were quantified using agar diffusion assays. Across all formulations and within each pH group, statistical analysis revealed that freeze-dried preparations generally produced larger inhibition zones than their spray-dried counterparts, consistent with the higher LC and more rapid short-term release associated with freeze-dried powders. Conversely, spray-dried microcapsules, despite their denser matrices and lower surface oil content, exhibited comparatively weaker diffusion-driven inhibition, in agreement with previous reports (Asres et al., 2025a). Importantly, the susceptibility profiles of *E. coli* and *L. innocua* were not uniform across all conditions. At neutral and alkaline pH, most formulations, with or without protease incorporation, yielded comparable inhibition-zone diameters against both bacteria, with enhanced activity against *L. innocua* only emerged in certain specific formulations. This observation aligns with previous findings by Brahmi et al. (2025b), who demonstrated that encapsulated carvacrol exhibits higher efficacy against Gram-positive bacteria relative to Gram-negative species, largely due to the outer membrane barrier of Gram-negative bacterial cells, such as *E. coli*, that restricts the penetration of hydrophobic phenolics like carvacrol. Under acidic conditions, protease treatment (particularly pepsin) markedly diminished the antibacterial activity of several spray-dried formulations against both *E. coli* and *L. innocua*. In contrast, the corresponding freeze-dried samples retained or modestly reduced their inhibitory capacity. This divergence can be attributed to the higher carvacrol LC of freeze-dried microcapsules and the core-material loss due to the emulsion destabilization caused by proteases treatment under pH 3.5. These findings indicate that although formulation variables such as pH and protease incorporation affect the morphology and encapsulation behavior of capsules, the resulting carvacrol microcapsules consistently preserve broad-spectrum antibacterial efficacy against both Gram-positive and Gram-negative bacteria, with particularly robust activity toward *Listeria*. Given the heightened sensitivity of *Listeria* to these microcapsules and its relevance as a persistent biofilm-forming pathogen in food-processing and healthcare environments, subsequent investigations focused exclusively on evaluating inhibition of planktonic *L. innocua* cells and eradication of its recalcitrant biofilms. A limitation of the present antibacterial evaluation is the absence of a blank PPI–maltodextrin microcapsule control without carvacrol or protease. Nevertheless, sunflower-oil microcapsules previously prepared using the same PPI–maltodextrin wall materials showed no detectable antibacterial activity under comparable conditions (data not shown), suggesting that the carrier matrix itself was unlikely to contribute substantially to the observed inhibition.

### 3.4 Minimal inhibitory concentrations (MIC) of microcapsules against *L. innocua* planktonic cells

MIC and MBC assays against *L. innocua* planktonic cells revealed marked dependence of antibacterial potency on drying technique, formulation pH, and protease incorporation (Table 5). Free carvacrol against *L. innocua* exhibited an MIC of 1.25 mg/mL in our preliminary experiments (data not shown). However, direct quantitative comparison with the microcapsule MIC values is not appropriate because the latter were expressed as total dry-powder mass concentration rather than carvacrol-equivalent concentration, and the formulations differed in loading capacity. Freeze-dried microcapsule formulations generally exhibited superior anti-*L. innocua* potency, with MIC as low as 1.25 mg mL^−1^, consistent with their higher carvacrol loading and enhanced short-term release. However, substantial variability was observed among freeze-dried microcapsules, particularly under acidic and neutral conditions. At pH 7, enzyme incorporation markedly increased MIC values (from 1.25 mg mL^−1^ in FN7 to 2.5 mg mL^−1^ in FP7 and 10 mg mL^−1^ in FT7), likely due to enzyme-mediated interfacial protein degradation, particularly trypsin, which compromise structural integrity and promoted core material loss during drying. A similar trend was noted in spray-dried powders, where the MIC increased from 2.5 mg mL^−1^ to 5 mg mL^−1^ for both SP7 and ST7 upon enzyme incorporation, aligning with the significantly reduced carvacrol loading observed in these samples. At pH 3.5, the MIC values of FP3.5 and FT3.5 decreased sharply from 2.50 to 1.25 mg mL^−1^, suggesting that specific hydrolysis products may facilitate rapid carvacrol availability upon rehydration, while the reconstituted but unstable emulsions likely generate larger oil droplets that further accelerate diffusion. In contrast, spray-dried powders generally showed slightly higher MIC (2.5–5 mg mL^−1^), related to their lower carvacrol loading, but they exhibited greater robustness against formulation-induced changes, particularly at pH 10. Furthermore, MBC values were generally consistent with, but higher than, the MIC values, indicating that bactericidal action requires a greater and more sustained exposure of carvacrol. Collectively, the antimicrobial efficacy of microcapsules is strongly dictated by their encapsulation characteristics (especially LC) and release kinetics, both of which depend on formulation stability.

**Table 5:** Effects of enzyme incorporation on the minimum inhibitory concentration (MIC) and the minimum bactericidal concentration (MBC) of protease-assisted pea protein–based microcapsules encapsulating carvacrol against planktonic *Listeria innocua* cells.

Drying technique	Microcapsules	MIC/(mg/mL)	MBC/(mg/mL)
Spray drying	SN7	2.50	10.00
	SP7	5.00	10.00
	ST7	5.00	10.00
	SN3.5	2.50	10.00
	SP3.5	5.00	10.00
	ST3.5	2.50	10.00
	SN10	2.50	5.00
	SP10	2.50	5.00
	ST10	2.50	10.00
Freeze drying	FN7	1.25	5.00
	FP7	2.50	5.00
	FT7	10.00	20.00
	FN3.5	2.50	10.00
	FP3.5	1.25	5.00
	FT3.5	1.25	5.00
	FN10	2.50	5.00
	FP10	2.50	5.00
	FT10	2.50	10.00

### 3.5 Antibiofilm activity of microcapsules against pre-formed *L. innocua* biofilms

This section primarily evaluates the ability of all carvacrol microcapsule formulations to eradicate *L. innocua* biofilms formed on polystyrene surfaces for 24 h. Particular emphasis was placed on the time-dependent (0.5 h, 1.0 h) and concentration-dependent (MIC, 1/2 MIC, 1/4 MIC) efficacy of these formulations against pre-formed *L. innocua* biofilms, with the aim of identifying the optimal microcapsule formulation and most effective concentration that delivers rapid and robust antibiofilm activity for potential applications in food processing and hygiene management. In addition, the effects of three factors, enzyme type (trypsin and pepsin), pH (3.5, 7, and 10), and drying technique (spray-drying and freeze-drying), on the antibiofilm performance of the microcapsules were systematically investigated. It should be noted that the present antibiofilm study included carvacrol-loaded microcapsules without protease as the main control but did not include a blank wall-material microcapsule or a free-carvacrol group. In our previous study, the antibiofilm activities of the free enzymes varied depending on both enzyme type and environmental pH. Free pepsin exhibited less than 50% biofilm inhibition under all tested conditions, whereas free trypsin showed substantially greater activity, achieving more than 60% inhibition at pH 10 and 6.250 μg/mL (Ji et al., 2025). Therefore, the primary objective was to evaluate whether protease incorporation could enhance the antibiofilm efficacy of carvacrol-loaded microcapsules.

#### 3.5.1 Eradication efficiency of pre-formed biofilms (EEB)

**Fig. 6:**
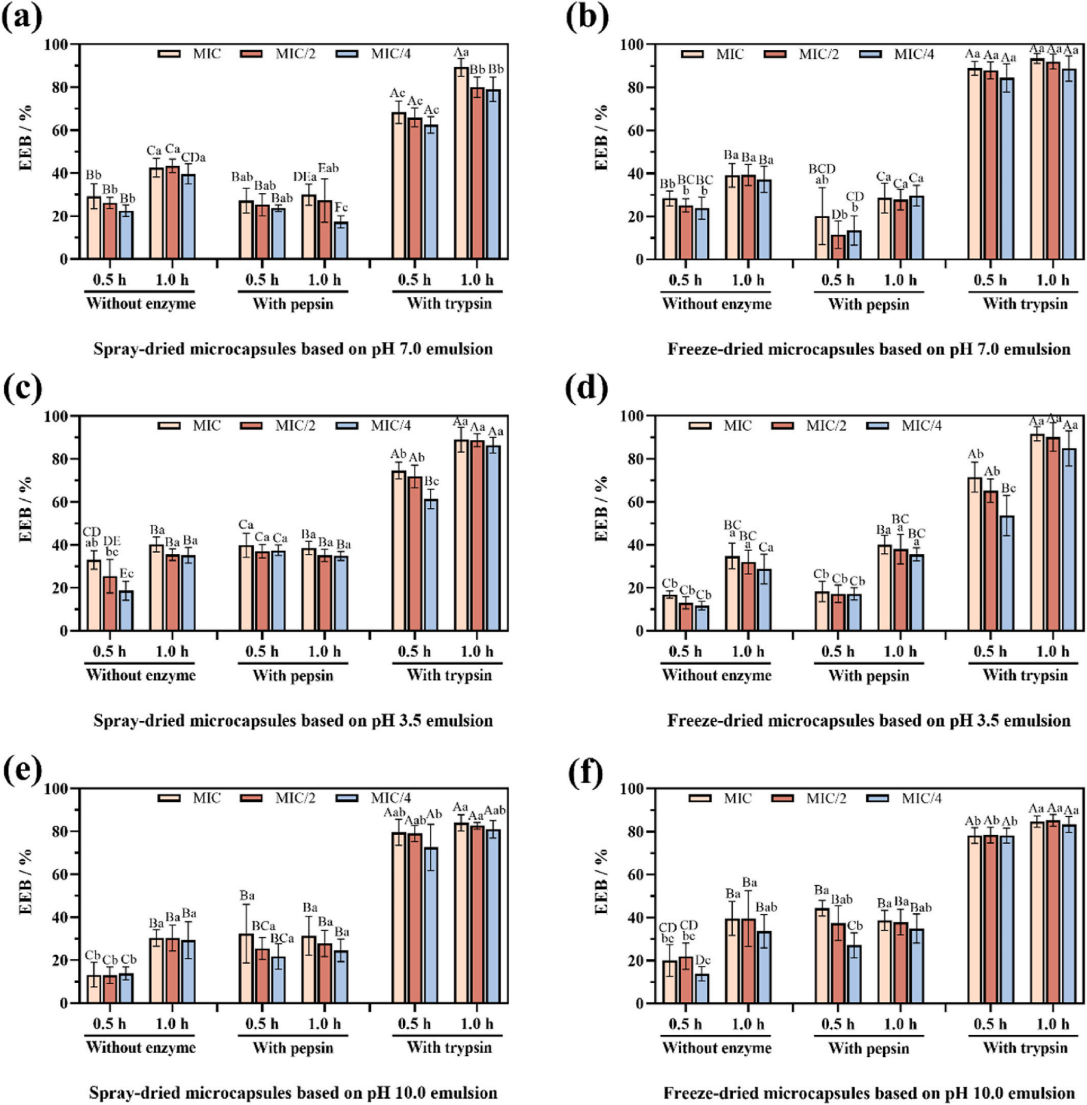
Effect of enzyme incorporation on the eradication of *L. innocua* biofilms formed on polystyrene surfaces by pea protein–based carvacrol microcapsules. (a, c, e) EEB of *L. innocua* biofilms after 0.5 h and 1 h of treatment with spray-dried microcapsules prepared at pH 7.0 (a), 3.5 (c), and 10.0 (e) using MIC, MIC/2, and MIC/4 concentrations; (b, d, f) EEB of *L. innocua* biofilms after 0.5 h and 1 h of treatment with freeze-dried microcapsules prepared at pH 7.0 (b), 3.5 (d), and 10.0 (f) using MIC, MIC/2, and MIC/4 concentrations. Different lowercase letters indicate significant differences among MIC levels and treatment time for the same capsule type. Different uppercase letters indicate significant differences between capsule types and MIC levels at same treatment time.

**Fig. 7:**
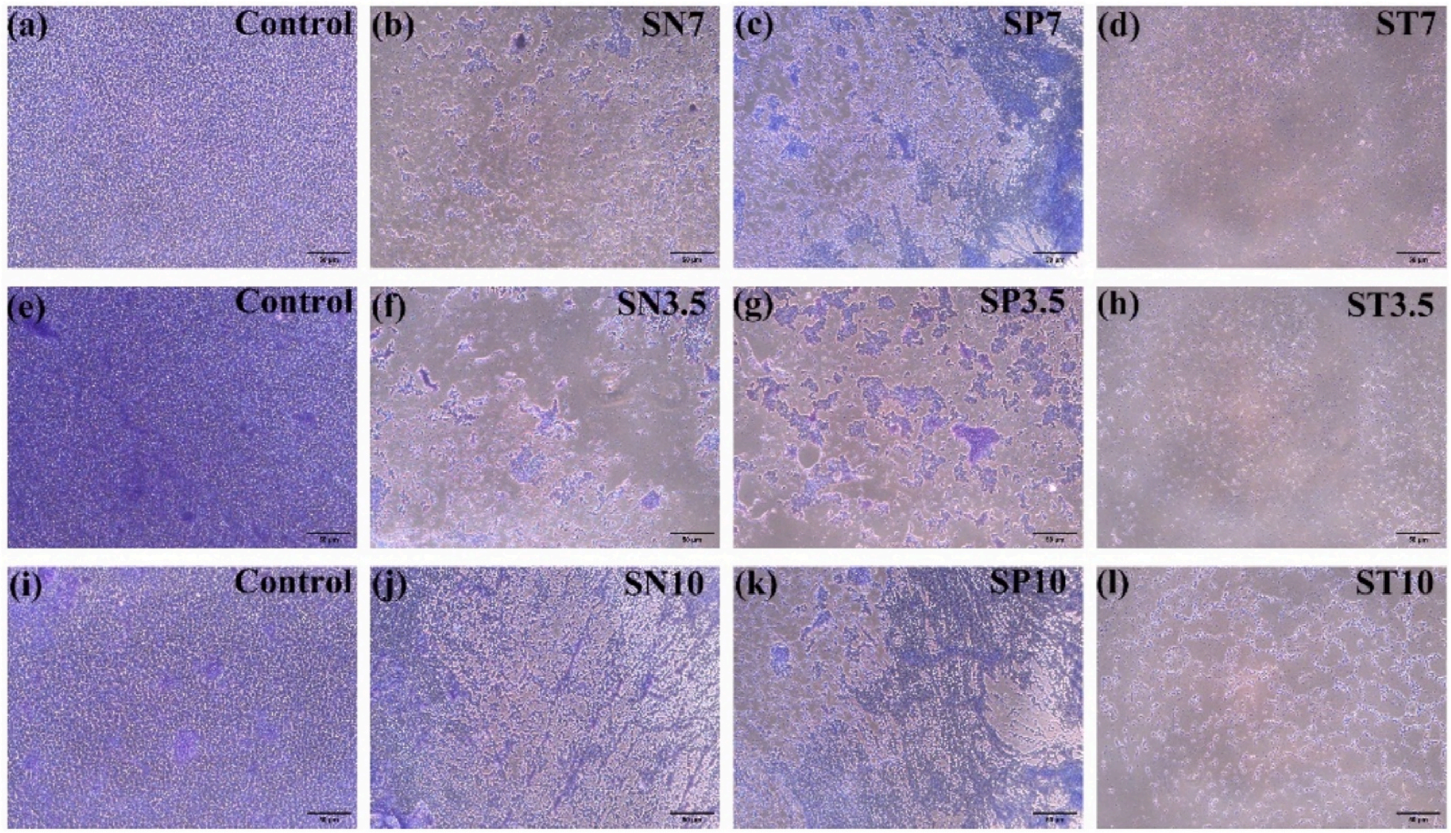
Microscopic observation of *L. innocua* biofilms after treatment with enzyme-loaded pea protein–based carvacrol spray-dried microcapsules. Representative optical micrographs of *L. innocua* biofilms formed on polystyrene surfaces after 1 h of exposure to microcapsules (at MIC) prepared at pH 7, 3.5, and 10, with and without incorporation of pepsin or trypsin. Scale bars = 50 μm.

**Fig. 8:**
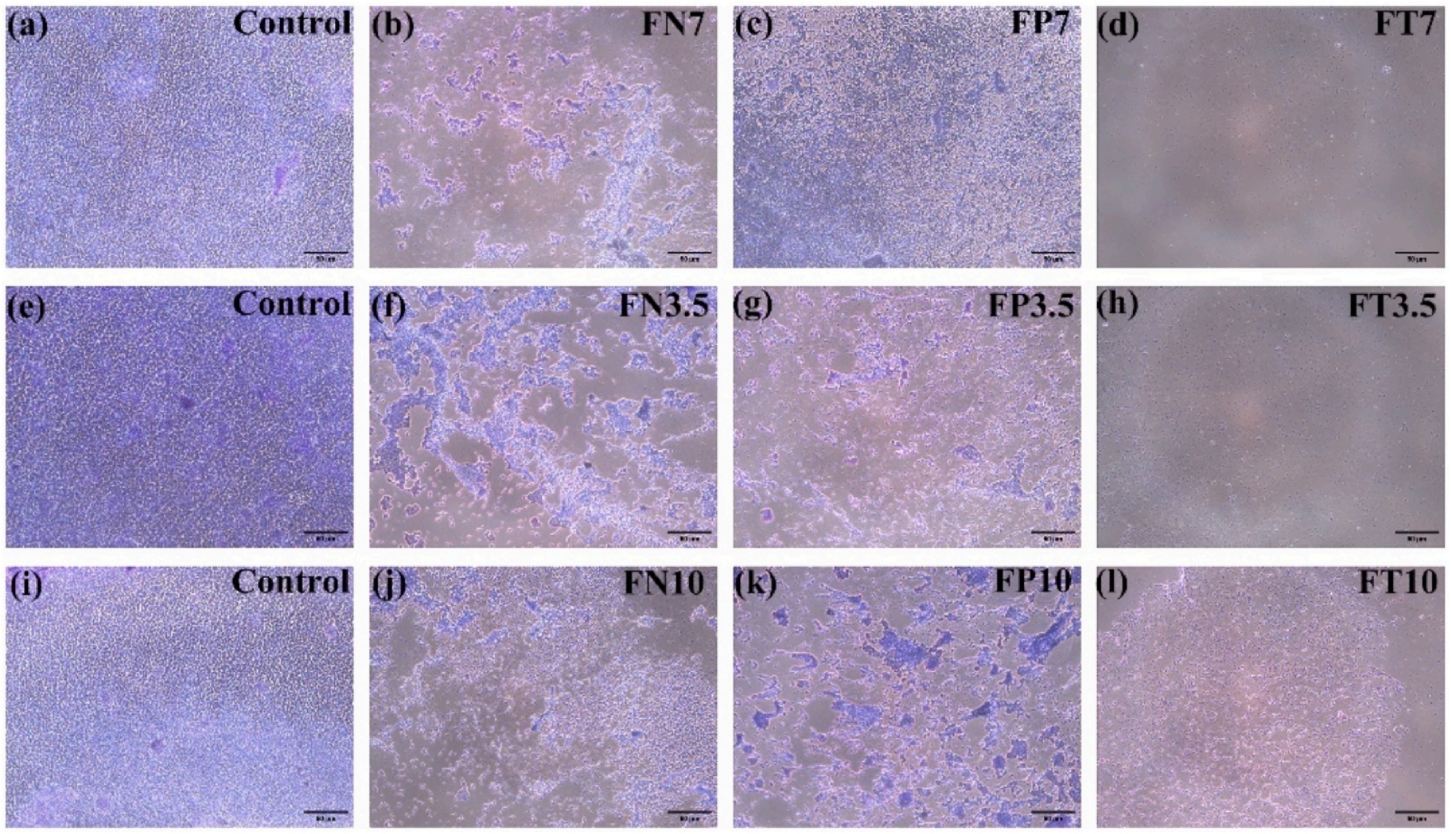
Microscopic observation of *L. innocua* biofilms after treatment with enzyme-loaded pea protein–based carvacrol freeze-dried microcapsules. Representative optical micrographs of *L. innocua* biofilms formed on polystyrene surfaces after 1 h of exposure to microcapsules (at MIC) prepared at pH 7, 3.5, and 10, with and without incorporation of pepsin or trypsin. Scale bars = 50 μm.

As shown in Fig. 6, all microcapsule formulations exhibited a clear time-dependent EEB against pre-formed *L. innocua* biofilms, whereas concentration-dependent effects appeared minimal. Regardless of the carvacrol concentration, longer exposure (1 h) consistently resulted in more rapid and pronounced biofilm eradication. This trend agrees with the findings of Zhang et al., who similarly reported no marked concentration-dependent biofilm dispersal within a broad antimicrobial range from 4MIC to MIC/16 (Zhang et al., 2022). It should be emphasized that the approximately 90% biofilm eradication measured by crystal violet staining corresponds to the removal of attached biofilm biomass rather than to a 90% reduction in viable biofilm-associated cells. In the present study, the term “biofilm eradication” therefore refers to the disruption, dispersion, and detachment of the preformed biofilm matrix from the polystyrene surface. This interpretation was further supported by the microscopic observations presented in Fig. 7, Fig. 8, which showed extensive disruption of the continuous biofilm network, reduced surface coverage, and large cleared areas after treatment with trypsin-assisted microcapsules.

*Effect of enzyme incorporation*. Across all microcapsule formulations, enzyme incorporation produced a striking and consistent pattern that the presence of trypsin, regardless of pH or drying method, significantly enhanced EEB, achieving approximately 90% after 1 h of treatment. Even at sub-MIC concentrations, trypsin-treated surfaces frequently reached nearly 80% eradication within short exposure periods, particularly at pH 7 and 10, highlighting the strong dispersive effect of proteolytic activity on biofilm structure. In line with this mechanism, Mechmechani et al. (2022a) reported that combined treatment with trypsin-loaded microcapsules and carvacrol microcapsules markedly reduced Gram-positive bacterial biofilms through enzymatic degradation of the EPS matrix. This enhancement is likely attributable to the strong proteolytic activity of trypsin, which hydrolyzes proteinaceous EPS components and weakens structural integrity, while simultaneously degrading pea protein within the microcapsule shell to increase the accessibility of encapsulated carvacrol to adherent cells. Conversely, despite maintaining high oil-loading, pepsin incorporation produced no notable improvement in EEB; even after 1 h at MIC, EEB values remained only 20-40%, comparable to, or slightly lower, than enzyme-free microcapsules (30-43%). This indicates that trypsin's specific cleavage sites and substrate affinity provide a more favorable preconditioning effect for biofilm dispersion than pepsin. These findings reinforce that combining proteases with EOs generates synergistic antibiofilm activity by accelerating membrane permeabilization and EPS disintegration in Gram-positive biofilms (Ghahari and Khosravi-Darani, 2024). Our results corroborate this synergy and further demonstrate that microencapsulation does not hinder protease-assisted penetration, particularly for trypsin, but instead enhances sustained release at the biofilm–liquid interface.

*Effect of formulation pH*. As shown in Fig. 6, the pH of the emulsions used to form the microcapsules exerted no significant overall effect on antibiofilm performance. This is likely because all microcapsules were dispersed in the same TS medium during EEB measurement, thereby attenuating or neutralizing pH-related differences. Nevertheless, microcapsules produced at pH 7.0 and 10.0 appeared to exert stronger short-term antibiofilm activity than those produced at pH 3.5. For example, freeze-dried microcapsules containing trypsin exhibited EEB values approximately 10-20% higher under neutral and alkaline conditions than under acidic conditions, with more consistent eradication at all concentrations. This may be attributed to enhanced microcapsule stability and more uniform shell formation based on neutral and alkaline emulsions, which improve carvacrol and enzyme retention and release kinetics, consistent with the earlier characterization results. Additionally, weaker performance at pH 3.5 may be related to the reported fact that acidic environments produce denser biofilm matrices that hinder molecular diffusion (Azeem et al., 2025). Besides, trypsin is active under neutral to alkaline conditions and preferentially hydrolyzes peptide bonds adjacent to Lys and Arg residues, which may increase its ability to disrupt susceptible proteinaceous domains within the *L. innocua* biofilm matrix. In contrast, pepsin requires acidic conditions for optimal activity and was likely less active after the powders were dispersed in the near-neutral TS treatment medium. Therefore, the better colloidal stability and encapsulation behavior of trypsin-assisted formulations at pH 7.0 and 10.0 may be advantageous for preservation of protease functionality and carvacrol accessibility after rehydration.

*Effect of drying technique*. Under neutral and alkaline conditions, freeze-dried microcapsules (Fig. 6b–d, f) appear to exhibit EEB values comparable to or higher than spray-dried samples (Fig. 6a–c, e), particularly under trypsin-assisted conditions where EEB frequently approached 90-99%. This enhanced performance may result from the higher carvacrol retention typically associated with freeze-dried powders, enabling more efficient diffusion after rehydration. Under acidic conditions, however, spray-dried microcapsules displayed equal or superior EEB (enzyme-free/pepsin-loaded, 19-40%; trypsin-loaded, 61-74%), compared with freeze-dried counterparts (enzyme-free/pepsin-loaded, 11-18%; trypsin-loaded, 53-71%). This may be attributed to the smaller specific surface area and reduced stability of freeze-dried capsules upon rehydration under acidic conditions, leading to less effective short-term release of carvacrol. In fact, the effect of drying technique may be related to the balance between active-compound retention and immediate accessibility after rehydration. Dense spray-dried particles can restrict premature carvacrol migration during storage, whereas porous freeze-dried matrices may provide shorter mass-transfer pathways and greater initial accessibility. This structure-dependent trade-off agrees with previous carvacrol–thymol delivery systems, in which wall architecture determined rapid initial availability versus more progressive release and persistent antibiofilm activity (Yammine et al., 2023b). Moreover, surface-engineered pMPC liposomes improved antibiotic transport into *P. aeruginosa* biofilms and produced greater eradication (60-65%) than PEGylated liposomes (45-50%) or the free drug (30-45%) (Kluzek et al., 2022), whereas co-spray-dried ciprofloxacin–alginate oligosaccharide powders demonstrated the feasibility of combining an antimicrobial with a biofilm-disrupting adjuvant in a storage-stable solid platform, although moisture sensitivity affected powder performance during storage (Zhang et al., 2024). These comparisons indicate that both biofilm accessibility and solid-state stability must be considered when evaluating the practical advantages of the present microcapsules.

In conclusion, these results demonstrate that trypsin-loaded pea protein-based carvacrol microcapsules exhibited the most effective antibiofilm activity against *L. innocua*, showing a marked time-dependent and a limited concentration-dependent response. MIC-level treatments for 1 h produced rapid and almost complete eradication, largely independent of formulation pH or drying method. The superior performance of trypsin-assisted formulations can be explained by the following points: first, trypsin retains greater proteolytic functionality under neutral to alkaline conditions, whereas pepsin requires an acidic environment and is expected to exhibit limited activity in the near-neutral TSB treatment medium. Second, trypsin preferentially cleaves peptide bonds adjacent to Lys and Arg residues and may therefore more effectively disrupt susceptible proteinaceous domains within the *Listeria* biofilm matrix. Third, trypsin-assisted nanoemulsions prepared at neutral and alkaline pH showed better colloidal stability and generated microcapsules with more favorable structural and encapsulation properties, thereby preserving both carvacrol availability and protease functionality. These findings highlight protease assistance as the dominant factor governing biofilm eradication by EO microcapsules, followed by secondary contributions from drying technique and formulation pH. The strong and rapid antibiofilm efficacy of these microcapsules underscores their potential for application on food-contact surfaces and in hygienic intervention strategies.

#### 3.5.2 Morphological observation of *L. innocua* biofilms after microcapsules treatment

Across all microscopic observations, the structural divergence among *L. innocua* biofilms subjected to different microcapsule treatments aligns closely with the biofilm eradication results. The control groups consistently exhibited a dense, uniform, and intensely stained closed woven chain network structure (Fig. 7a–e, i; Fig. 8a–e, i), a typical characteristic of mature *Listeria* biofilm matrix, consistent with the findings of Wang et al. (2022b). In the enzyme-free and pepsin-loaded microcapsule groups (SN, SP, FN, FP in Fig. 7, Fig. 8), only partial structural disruption was observed. The biofilm became irregular and loosely structured, displaying extensive fissures and fragmented aggregates, but large contiguous regions of intact matrix persisted, consistent with their approximately 40% EEB. This disruption was weakest in pepsin-loaded microcapsules at pH 7 and 10, where abundant sheet-like, purple-stained clusters remained, comparable to the enzyme-free samples. These observations indicated that incorporating pepsin conferred limited enhancement of antibiofilm activity. This aligns with findings by Mechmechani et al. (2023), who reported minimal structural alteration in Gram-positive *Enterococcus faecalis* biofilms following pepsin treatment, and noted a weaker synergistic elimination effect when combined with carvacrol compared with Gram-negative biofilms. In contrast, trypsin-loaded microcapsules (ST, FT in Fig. 7, Fig. 8) induced profound structural collapse of the mature biofilm, nearly eliminating the visible matrix in accordance with their >90% eradication rates. Across all pH conditions, the biofilms exhibited extensive matrix degradation, markedly reduced staining, and the emergence of large cleared zones, with only thin residual microbial layers detectable. These features reflected substantial EPS decomposition and weakened cellular adhesion. Collectively, the images corroborate that trypsin-loaded carvacrol microcapsules, irrespective of pH or drying method, maintain exceptional efficacy in eradicating mature biofilms of Gram-positive *L. innocua*, and highlight the robust synergistic mechanism between trypsin and carvacrol within the encapsulated system. However, the use of a single strain, surface material, biofilm maturation period, and treatment regime limits the direct extrapolation of the results to food-processing environments. Further validation of the selected trypsin-assisted formulations on stainless steel and other food-contact materials, under different biofilm ages and environmental conditions, is therefore required.

### 3.6 Antibacterial and antibiofilm activity of microcapsules after storage at 4 °C for one year

After one year of storage at 4 °C, the MIC results indicated that freeze-dried microcapsules experienced the most pronounced loss of antibacterial potency, particularly those formulated at pH 3.5 (Table 6). In these acidic systems, the MIC values of the enzyme-loaded powders exhibited substantial increases, for both trypsin and pepsin, with the MIC of the lyophilized powder increasing four to eight times (FP3.5, 1.25 → 10 mg/mL; FT3.5: 1.25 → 5 mg/mL). These changes suggested extensive degradation of the wall matrix during storage and diminished carvacrol retention, consistent with the structural instability of enzyme-loaded microcapsules formed under acidic conditions. In contrast, spray-dried microcapsules showed considerably smaller MIC shifts, in agreement with their more uniform and structurally stable spherical morphology, as well as good encapsulation properties. Microcapsules prepared at pH 7 and 10 demonstrated the greatest storage stability, showing minimal or no MIC increase, especially in enzyme-loaded formulations in which the MIC remained unchanged. These trends align with earlier physicochemical characterization: emulsions at neutral and alkaline pH yielded cohesive wall structures with superior EE and controlled-release behavior, resulting in slower volatility loss of carvacrol; whereas acidic emulsions produced weaker matrices with higher proportions of surface-associated, poorly retained oil prone to leakage during long-term storage.

**Table 6:** Changes in the minimum inhibitory concentration (MIC) of protease-assisted pea protein-based microcapsules encapsulating carvacrol against planktonic *Listeria innocua* after one year of storage at 4 °C.

Drying technique	Microcapsules	MIC/(mg/mL) Before → After storage	Drying technique	Microcapsules	MIC/(mg/mL) Before → After storage
Spray drying	SN7	2.50→5.00	Freeze drying	FN7	1.25→2.50
	SP7	5.00→5.00		FP7	2.50→2.50
	ST7	5.00→5.00		FT7	10.00→10.00
	SN3.5	2.50→5.00		FN3.5	2.50→2.50
	SP3.5	5.00→10.00		FP3.5	1.25→10.00
	ST3.5	2.50→10.00		FT3.5	1.25→5.00
	SN10	2.50→5.00		FN10	2.50→5.00
	SP10	2.50→2.50		FP10	2.50→2.50
	ST10	2.50→2.50		FT10	2.50→2.50

**Fig. 9:**
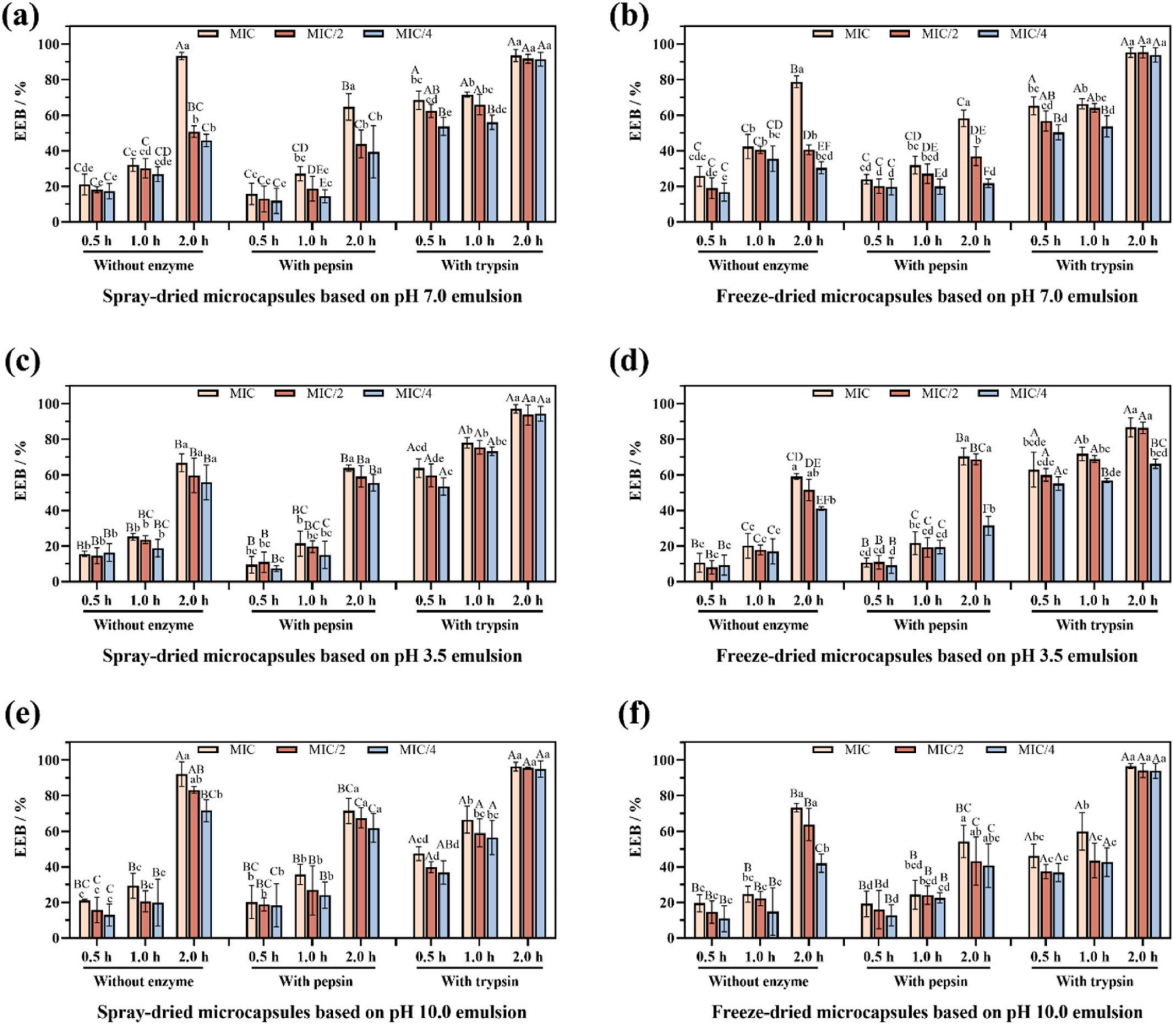
Eradication activity of pea protein-based carvacrol microcapsules after one year of storage against *L. innocua* biofilms formed on polystyrene surfaces. (a, c, e) EEB of *L. innocua* biofilms after 0.5 h, 1 h and 2 h of treatment with spray-dried microcapsules prepared at pH 7.0 (a), 3.5 (c), and 10.0 (e) using MIC, MIC/2, and MIC/4 concentrations; (b, d, f) EEB of *L. innocua* biofilms after 0.5 h, 1 h and 2 h of treatment with freeze-dried microcapsules prepared at pH 7.0 (b), 3.5 (d), and 10.0 (f) using MIC, MIC/2, and MIC/4 concentrations. Different lowercase letters indicate significant differences among MIC levels and treatment time for the same capsule type. Different uppercase letters indicate significant differences between capsule types and MIC levels at same treatment time.

Consistent with the decline in antibacterial activity against planktonic *L. innocua*, the antibiofilm efficacy of aged microcapsules also decreased modestly for most formulations, most notably for enzyme-free and pepsin-loaded systems (Fig. 9). These formulations exhibited reduced early-phase eradication (within 1 h) and lower maximal EEB values. Nevertheless, the overall activity remained strongly time-dependent, and the relative performance ranking was preserved. A consistent pattern across all pH values and drying methods was that trypsin-loaded microcapsules retained the highest antibiofilm performance after storage, still achieving near-complete eradication at extended exposure times, with EEB values of 85–99%. This outcome suggested that the proteolytic synergy provided by trypsin remains largely intact despite prolonged storage. pH-dependent differences remained subtle, although microcapsules produced at pH 7 and 10 maintained stronger antibiofilm activity than those prepared at pH 3.5. For example, pepsin-loaded acidic microcapsules showed ≤21% EEB at 1 h, whereas neutral and alkaline formulations reached 25–35%, all lower than their pre-storage EEB values and consistent with the observed MIC increase, indicating reduced carvacrol after storage. Drying-method effects remained minor after storage, though a reversal was observed. Enzyme-free and pepsin-loaded spray-dried microcapsules typically achieved maximal EEB values 10–20% higher than their freeze-dried counterparts across all pH conditions. This was possibly due to the superior long-term oil retention afforded by their spherical microstructure. For trypsin-loaded samples, drying method had negligible impact post-storage, as their eradication efficiency reached a plateau within 2 h regardless of processing route. Among these formulations, the partial activity loss may also involve protease inactivation and physical aging of the PPI–maltodextrin wall matrix. During prolonged storage, residual moisture may increase matrix mobility and promote structural relaxation and carvacrol migration, while enzyme unfolding, aggregation, or oxidation may reduce recovered proteolytic activity after rehydration (Chen et al., 2021; Xiao et al., 2022; Weng et al., 2024). These effects could be minimized through lower water activity, moisture- and oxygen-barrier packaging, and the incorporation of enzyme-protective sugars. Direct monitoring of residual protease activity and wall-matrix aging during storage will be required to distinguish these contributions.

Overall, long-term refrigerated storage caused moderate reductions in both antibacterial and antibiofilm functions across all formulations; however, trypsin-assisted microcapsules maintained a high degree of functional integrity. These findings reinforce the dominant role of protease-assisted mechanisms in biofilm eradication, and highlight pH 7 and pH 10 formulations as the most robust candidates for applications requiring long-term functionality.

## 4 Conclusion

This study established a clear mechanistic link between enzyme-modulated nanoemulsion stability, pea protein-based maltodextrin microcapsule performance, and the resulting antibacterial and antibiofilm efficacy of encapsulated carvacrol. Trypsin-incorporated emulsions at neutral-alkaline pH produced finer, more charged droplets whereas pepsin provoked pronounced destabilization under acidic conditions. Upon microencapsulation with maltodextrin, drying method emerged as the primary determinant of powder architecture and encapsulation behavior: (**i**) spray-drying generated dense, spherical particles with low surface oil (0.95-4.49 mg g^−1^) and very high encapsulation efficiencies (>96–99%), with lower loading capacities (57–141 mg g^−1^) and some volatility losses during processing; (**ii**) freeze-drying afforded markedly higher loading capacity and retention (LC up to ≈238 mg g^−1^ and RE up to ≈96%), but produced porous, fragile matrices with elevated surface oil and reduced EE, especially when emulsions were destabilized (notably at pH 3.5 and incorporated with pepsin). ATR-FTIR confirmed that the maltodextrin matrix dominated spectra and that carvacrol signals were largely suppressed in well-encapsulated samples, whereas acidic, enzyme-incorporated formulations displayed weakened amide bands and re-emergent carvacrol peaks, consistent with interfacial erosion and core migration.

Functionally, antibacterial and antibiofilm outcomes were governed principally by encapsulated carvacrol loading and protease assistance. Trypsin-loaded microcapsules combined enhanced interfacial packing and proteolytic disruption of proteinaceous EPS to deliver the most potent and rapid antibiofilm activity (≈90% biofilm eradication within 1 h and MIC as low as 1.25 mg mL^−1^ in optimal formulations), effects that were preserved after 12 months at 4 °C (EEB ≈85–99% within 2 h treatment). In contrast, pepsin impaired structural integrity and long-term performance, particularly in acidic systems where MICs of lyophilized powders increased substantially (e.g., FP3.5, from 1.25 to 10 mg mL^−1^), also exhibiting low biofilm eradication comparable to enzyme-free formulations (20-40%). Overall, these findings highlight that rational control of protease type, formulation pH, and drying method can be used to tune interfacial assembly, encapsulation behavior, and antimicrobial and antibiofilm outcomes. Trypsin-assisted microcapsules produced at neutral or alkaline pH constitute promising candidates for further validation in food-safety and surface-hygiene applications. However, the present study was limited to laboratory-scale processing and an in vitro *L. innocua* biofilm model on polystyrene surfaces. Further studies are needed to evaluate process scale-up, carvacrol-release behavior, residual enzyme activity, structural changes during storage, and performance under realistic food and food-contact conditions.

## Ethics approval and consent to participate

Not applicable. This study did not involve human participants, human data, or animals.

## Consent for publication

Not applicable.

## Data availability

The data supporting the findings of this study are included in the article. Further inquiries can be directed to the corresponding author.

## Funding

This research did not receive any specific grant from funding agencies in the public, commercial, or not-for-profit sectors.

## Declaration of competing interest

The authors declare that they have no known competing financial interests or personal relationships that could have appeared to influence the work reported in this paper.
